# PLCγ2 regulates TREM2 signalling and integrin-mediated adhesion and migration of human iPSC-derived macrophages

**DOI:** 10.1038/s41598-021-96144-7

**Published:** 2021-10-06

**Authors:** Juliane Obst, Hazel L. Hall-Roberts, Thomas B. Smith, Mira Kreuzer, Lorenza Magno, Elena Di Daniel, John B. Davis, Emma Mead

**Affiliations:** 1 Alzheimer’s Research UK Oxford Drug Discovery Institute, Centre for Medicines Discovery, University of Oxford, Oxford, UK; 2 James Martin Stem Cell Facility, Sir William Dunn School of Pathology, University of Oxford, Oxford, UK; 3 Department of Oncology, MRC Weatherall Institute of Molecular Medicine, University of Oxford, Oxford, UK; 4 UCL Alzheimer’s Research UK Drug Discovery Institute, London, UK; 5 Present Address: UK Dementia Research Institute (UK DRI) At Cardiff University, Cardiff, UK; 6 Present Address: Institute of Pathology, University of Munich, Munich, Germany; 7 Present Address: Astex Pharmaceuticals, Cambridge, UK

**Keywords:** Cell biology, Immunology, Neuroscience, Stem cells

## Abstract

Human genetic studies have linked rare coding variants in microglial genes, such as *TREM2*, and more recently *PLCG2* to Alzheimer’s disease (AD) pathology. The P522R variant in *PLCG2* has been shown to confer protection for AD and to result in a subtle increase in enzymatic activity. PLCγ2 is a key component of intracellular signal transduction networks and induces Ca^2+^ signals downstream of many myeloid cell surface receptors, including TREM2. To explore the relationship between PLCγ2 and TREM2 and the role of PLCγ2 in regulating immune cell function, we generated human induced pluripotent stem cell (iPSC)- derived macrophages from isogenic lines with homozygous *PLCG2* knockout (Ko). Stimulating TREM2 signalling using a polyclonal antibody revealed a complete lack of calcium flux and IP1 accumulation in PLCγ2 Ko cells, demonstrating a non-redundant role of PLCγ2 in calcium release downstream of TREM2. Loss of PLCγ2 led to broad changes in expression of several macrophage surface markers and phenotype, including reduced phagocytic activity and survival, while LPS-induced secretion of the inflammatory cytokines TNFα and IL-6 was unaffected. We identified additional deficits in PLCγ2- deficient cells that compromised cellular adhesion and migration. Thus, PLCγ2 is key in enabling divergent cellular functions and might be a promising target to increase beneficial microglial functions.

## Introduction

Alzheimer’s disease (AD) is a chronic neurodegenerative disease and the most common cause of dementia. Neuropathological features of AD typically include the accumulation of β-amyloid in extracellular plaques, neurofibrillary tangles composed of hyperphosphorylated tau protein, and the occurrence of synaptic and neuronal loss that leads to progressive cognitive impairment. There is a strong genetic component in disease aetiology, with an estimated heritability of 58 – 79% in late-onset AD (LOAD)^1^. Recent large-scale genetic studies have identified an increasing number of susceptibility genes linked to conferring either risk or protection in developing LOAD. Many of these genes are predominantly expressed in myeloid cells such as microglia, the brain’s resident immune cells, emphasizing the role of the innate immune response in AD pathogenesis. Amongst the polymorphisms discovered are rare coding variants in genes encoding Triggering Receptor Expressed on Myeloid cells 2 (TREM2) and Phospholipase Cγ2 (PLCγ2)^2–4^. The missense variant P522R in *PLCG2* (rs72824905-G, *P* = 5.38 × 10^−10^, OR = 0.68) was shown to be protective, not only in the context of AD^4,5^, but also in other neurodegenerative diseases such as frontotemporal dementia and dementia with Lewy bodies^6^.

PLCγ2 is a signalling enzyme activated through tyrosine phosphorylation by receptor and non-receptor kinases^7^. It hydrolyses the membrane phospholipid phosphatidylinositol 4,5-bisphosphate (PIP2) to the secondary messengers inositol 1,4,5-trisphosphate (IP3) and diacylglycerol (DAG). IP3 binds to ligand-gated ion channels present in the endoplasmic reticulum, increasing intracellular Ca^2+^ levels. DAG remains bound to the membrane and activates protein kinase C (PKC) and Ras guanyl-nucleotide-releasing proteins (RasGRPs), which initiate Nuclear Factor-kappa B (NF-κB) and mitogen-activated protein kinase (MAPK) pathways. PLCγ2 shares high structural and functional similarities with PLCγ1^8^. While PLCγ1 is ubiquitously expressed and regulates growth factor signalling in a variety of cell types, expression of PLCγ2 is largely restricted to hematopoietic cells, and regulates specific responses downstream of several immune receptors^8^. As such, PLCγ2 plays a critical role in B cell receptor signalling and is involved in cell-specific function of platelets, mast cells, NK cells and macrophages by regulating Fc-receptor signalling^9,10^.

A number of pathogenic mutations in PLCγ2 have emerged that were shown to be implicated in autoimmune disease and cancer. The first indication of its role in autoimmune disease was revealed in mutagenesis screens in mice, identifying gain-of function mutations in PLCγ2. The mouse strains abnormal limb -5 and -14 (Ali5 and Ali14) harbour single amino acid substitutions in PLCγ2 that result in severe spontaneous inflammation and autoimmunity^11,12^. In human genetic studies, dominantly inherited immune disorders have been linked to germline mutations in PLCγ2. These mutations are enriched in affected families and localise to the autoinhibitory regulatory domains of the enzyme, resulting in constitutively increased activity of PLCγ2. Resulting syndromes manifested in these patients have been named PLAID (PLCγ2-associated antibody deficiency and immune dysregulation), with key symptoms being cold-induced urticaria, antibody deficiency, and susceptibility to infection and autoimmunity^13^, and APLAID (autoinflammatory PLAID), which is characterized by skin lesions, bronchiolitis, arthralgia, ocular inflammation, enterocolitis, absence of autoantibodies and mild immunodeficiency^14^. Interestingly, while hypermorphic APLAID-linked mutation (S707Y substitution) leads to increased downstream signalling, the deletion mutations associated with PLAID resulted in reduced downstream signalling evidenced by reduced Ca^2^^+^ flux and MAPK activation in B cells and NK cells, pointing to potentially distinct and highly complex effects on cellular signalling and feedback mechanisms^15^.

The genetic association of the P522R variant with AD was initially discovered in 2017^4^ and since then re-confirmed in independent cohorts^5,16^, and was also shown to be linked to mitigated tau pathology, reduced cognitive decline and longevity^17^. Similar to the PLAID and APLAID mutations, this protective variant is located in the autoinhibitory regulatory region of PLCγ2, and has a slight hypermorphic effect on enzymatic function in recombinant cell lines^18^. Expression of P522R in mouse bone marrow-derived macrophages (BMDM) resulted in increased PLCγ2 activity and promoted survival, *E.coli* phagocytosis and LPS response^19^. Additionally, P522R showed increased enzymatic activity when expressed in human iPSC-derived microglia-like cells, as well as in mouse microglia and macrophages, evidenced by increased Ca^2+^ release and reduced PIP2 levels upon Fc-receptor ligation^20^. In contrast to the previous observations in BMDMs, P522R led to reduced phagocytosis of fungal and bacterial particles in the cell models used in this study, while endocytosis of oligomeric Aβ_42_ was enhanced^20^.

TREM2 is a cell-surface receptor of the immunoglobulin superfamily implicated in a variety of microglial functions, such as phagocytosis, chemotaxis and survival^21–23^. TREM2 associates with the immunoreceptor tyrosine-based activation motif (ITAM)–containing adaptor protein DAP12 to induce signal transduction, including recruitment and activation of spleen tyrosine kinase (SYK) and calcium flux^24,25^. TREM2 belongs to the same interaction network of immune-response genes implicated in AD as PLCγ2^4^ and has been recently shown to signal through PLCγ2 to mediate cell survival, phagocytosis, processing of neuronal debris and lipid metabolism^26^.

In this study we aimed to characterize in detail the role of PLCγ2 as a critical effector of several functional phenotypes in a human iPSC model of microglia (iPSC-derived macrophages), including the previously unexplored impact of PLCγ2 deficiency on cell adhesion and migration. Studying microglia using human cell models such as iPSC-derived cells is vital given the species-specific differences in microglial responses between human and mouse cells^27^. We observed a complete loss of Ca^2+^ signal induced by specific TREM2 ligation in PLCγ2-deficient cells. The absence of PLCγ2 resulted in changes in expression of several macrophage receptors and reduced TREM2-dependent phagocytic activity and survival, emphasizing the importance of PLCγ2 for establishing TREM2-mediated key myeloid functions. We demonstrate that the lack of PLCγ2 led to deficits in cellular adhesion and migration in human iPSC-macrophages, possibly via integrin-dependent mechanisms. The results shown here indicate that PLCγ2 is important to facilitate a variety of cellular responses. This further supports the hypothesis that modifying PLCγ2 may be a potential therapeutic strategy to manipulate microglial functions in Alzheimer’s disease.

## Results

### PLCγ2 is activated upon TREM2 ligation and modulates calcium signal downstream of TREM2

Human iPSC-derived microglia-like cells are an authentic and relevant model to study microglial function and phenotypes in vitro. We generated primitive, tissue-type macrophages differentiated from human iPSC following a well-established protocol^28^. These cells closely resemble human foetal microglia with regards to transcriptional signature and express high levels of microglial genes^29,30^.

**Figure 1:**
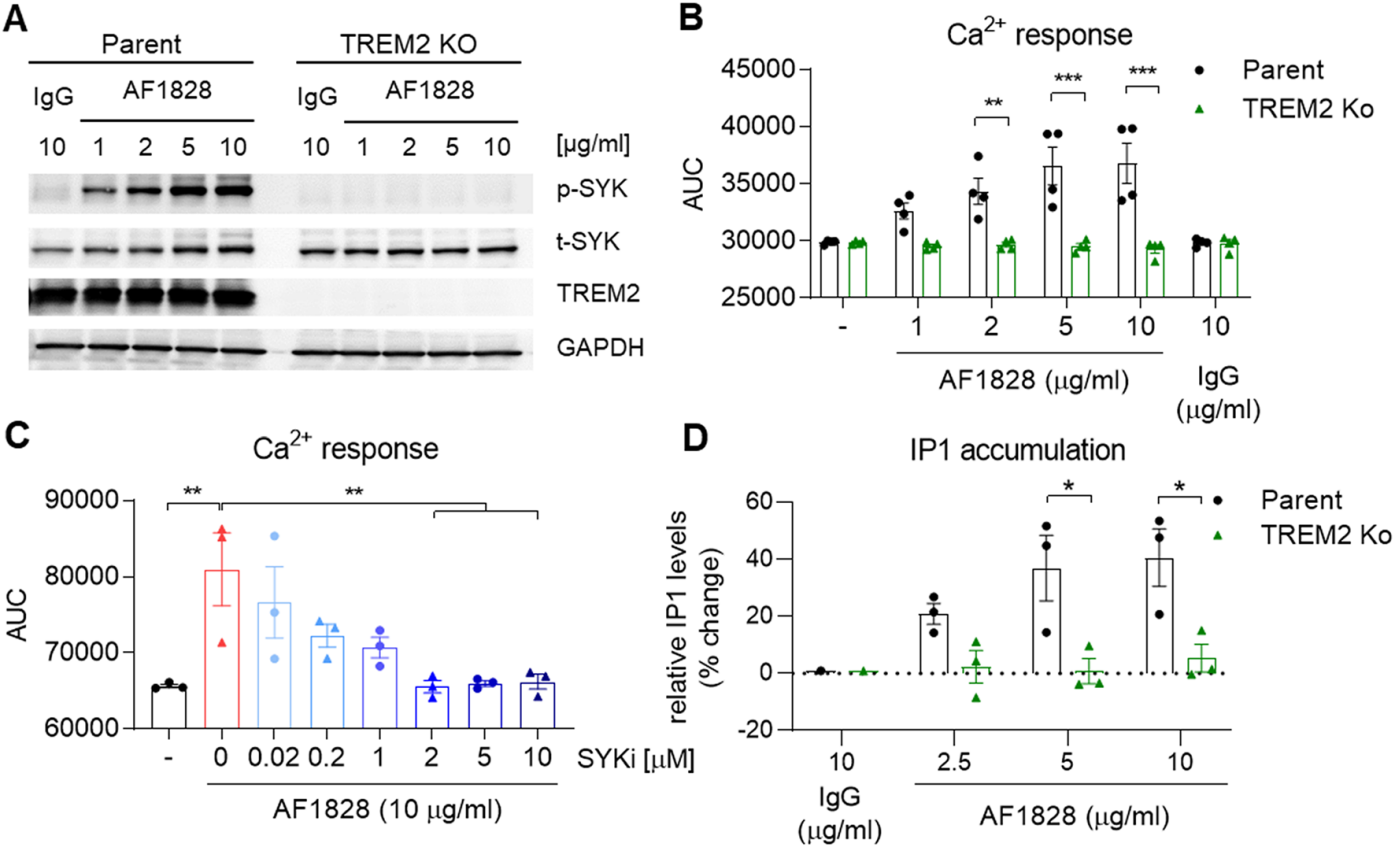
TREM2 agonism induces PLC activity in iPSC- derived macrophages. (**A**) Representative Western blot showing phosphorylation of SYK after TREM2 stimulation for 5 min using a TREM2-specific antibody (AF1828, R&D Systems) in Parent and TREM2 Ko cells. Full immunoblot images are presented in Supplementary Fig. 6. (**B**) Ca^2+^ flux induced by TREM2 ligation in Parent cells is absent in TREM2 Ko cells. n = 4. (**C)** SYK inhibitor BIIB-057 (SYKi) reduces TREM2 antibody-evoked Ca^2+^ signal in a dose-dependent manner in Parent cells. n = 3. (**D)** TREM2-induced IP1 accumulation determined by HTRF assay is prevented in TREM2 Ko cells. n = 3, data shown represent mean ± SEM, (**B**, **D**) two-way ANOVA followed by Bonferroni’s multiple comparison test, (**C**) one-way ANOVA followed by Bonferroni’s multiple comparison test, *p < 0.05, **p < 0.01, ***p < 0.001.

In this study, we aimed to investigate the role of PLCγ2 within the TREM2 pathway and its effect on macrophage phenotypes. In order to stimulate TREM2 we used a TREM2 activating antibody (R&D systems, AF1828) that induces downstream signalling including SYK activation and release of calcium from intracellular stores^23,31^. We observed concentration-dependent SYK phosphorylation upon TREM2 antibody stimulation in the wild-type BIONi010-C (Parent) line, which was absent in the isogenic TREM2 Ko line (Fig. 1A), confirming TREM2 specificity of the antibody. PLCγ2 enzymatic activity hydrolyses the membrane phospholipid PIP2 into IP3 and DAG, which leads to Ca^2+^ flux and PKC activation. To determine PLC activity upon TREM2 activation, we measured intracellular Ca^2+^ signal in iPSC macrophages when stimulated with AF1828. Parent cells showed a robust Ca^2+^ response upon stimulation, which was absent in the TREM2 Ko cells (Fig. 1B, Suppl. Figure 3A). Although a TREM2-mediated Ca^2+^ response was absent, TREM2 Ko cells showed normal calcium kinetics upon addition of ATP (Suppl. Figure 3B). The TREM2 Ca^2+^ signal was dependent on SYK signalling, as pre-incubation with the SYK inhibitor BIIB-057 reduced the signal in a dose-dependent manner (Fig. 1C, Suppl. Figure 3D), indicating that PLC-evoked calcium ion release is downstream of SYK. We further detected inositol monophosphate (IP1), a stable downstream metabolite of IP3 generated by activation of PLC using an HTRF assay. We observed IP1 accumulation in Parent cells when stimulated with AF1828 in a concentration-dependent manner, while TREM2 Ko cells failed to accumulate IP1, further validating the lack of PLC response to AF1828 in the absence of TREM2 (Fig. 1D).

**Figure 2:**
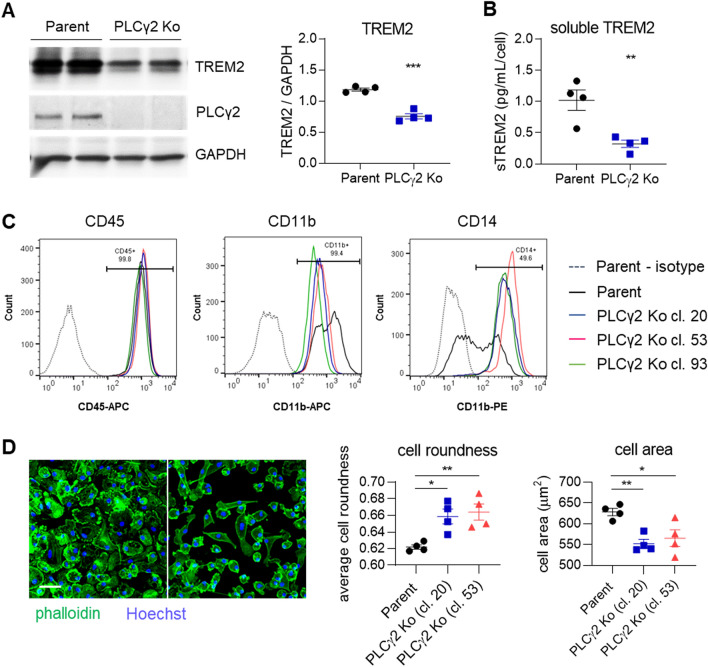
PLCγ2 Ko leads to dysregulation of cell surface marker expression and morphological changes in iPSC-derived macrophages. (**A**) Western blot confirms lack of PLCγ2 protein in PLCγ2 Ko and shows reduction in TREM2 expression in PLCγ2 KO macrophages compared to Parent. n = 4. Full immunoblot images are presented in Supplementary Fig. 7. (**B**) Levels of soluble TREM2 detected in cell supernatant 8 days after plating are lower in PLCγ2 Ko cells compared to Parent. n = 4, (**C**) Macrophage surface markers CD11b, CD14, and CD45 were measured by flow cytometry, compared to relevant isotype IgG. Annotations indicate frequency of marker positivity in the Parent line. (**D)** Morphology of macrophage lines was determined by phalloidin staining and analysis of cell roundness and cell area. Scale bar 50 μm. n = 4, data shown represent mean ± SEM, One-way ANOVA followed by Bonferroni’s multiple comparison test. *p < 0.05, **p < 0.01.

To study the function of PLCγ2 in TREM2-mediated responses, homozygous PLCγ2 Ko iPSC lines were generated in the wild-type BIONi010-C line. Three Ko clones were selected that showed no major genetic alterations determined by Illumina SNP microarray analysis (Suppl. Figure 1) and were validated for the absence of PLCγ2 protein by Western blotting (Suppl. Figure 2A). Parent and PLCγ2 Ko lines showed similar amounts of *PLCG1* gene expression, as determined by RNAscope analysis, excluding the possibility that PLCγ1 is dysregulated to compensate for the absence of PLCγ2 (Suppl. Figure 2B). We analysed TREM2 expression in the PLCγ2 Ko clones and observed reduced TREM2 levels compared to Parent iPSC-macrophages, determined by Western blot (Fig. 2A, Suppl. Figure 2A). This finding has to be kept in mind as a lower abundance of TREM2 on the cell surface could account for changes in cell signalling and phenotype. The decrease in total TREM2 protein also results in a lower degree of TREM2 being shed from the membrane, evidenced by lower levels of soluble TREM2 detected in the supernatant (Fig. 2B). We validated macrophage identity after differentiation of the PLCγ2 Ko iPSC lines by detection of typical lineage markers by flow cytometry, which were expressed in all lines (Fig. 2C). The three PLCγ2 Ko clones however showed lower surface expression of CD11b, while CD14 levels were clearly increased, possibly indicating differences in basal cell activation in the absence of PLCγ2. iPSC-macrophages differentiated from PLCγ2 Ko clones also demonstrated differences in morphology, signified by higher cell roundness and smaller cell area (Fig. 2D). The finding that baseline expression of several macrophage surface markers as well as cell morphology are distinctly altered in PLCγ2 Ko cells points to fundamental differences in cells differentiated in the absence of PLCγ2 that could lead to altered functional effects observed in the Ko lines. As we found similar responses of the three clones in all assays we conducted, we hereafter show representative data from clone 20 when not otherwise stated.

**Figure 3:**
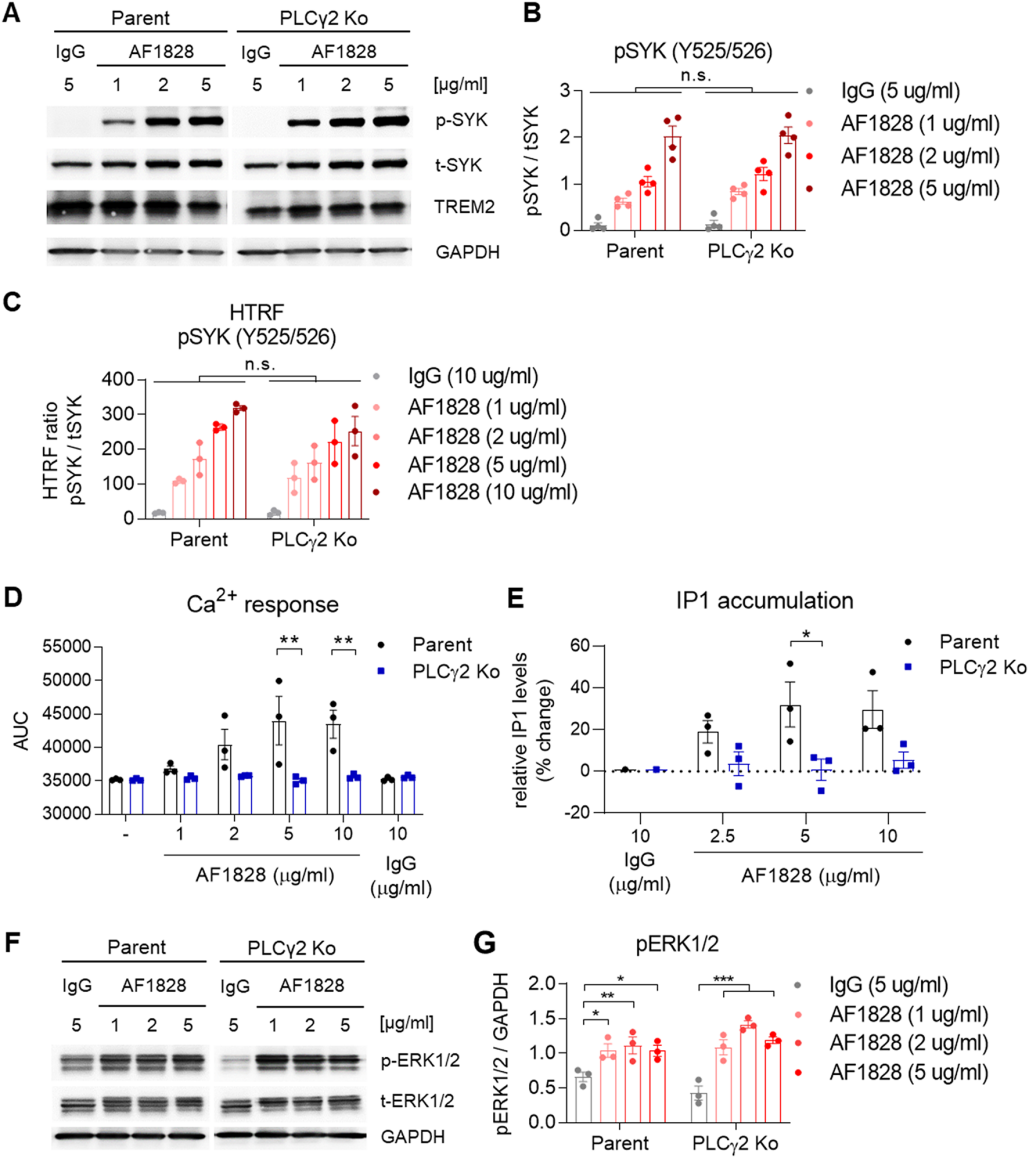
PLCγ2 regulates TREM2-mediated signalling in iPSC- derived macrophages. (**A**) Representative Western blots showing phospho-SYK after TREM2 ligation using AF1828 in Parent and PLCγ2 Ko cells. (**B**) Quantification of SYK phosphorylation after TREM2 ligation shows no effect of PLCγ2 deficiency, n = 4. Full immunoblot images are presented in Supplementary Fig. 9. (**C**) pSYK HTRF assay confirms similar phosphorylation levels upstream of PLCγ2 upon TREM2 stimulation in Parent and PLCγ2 Ko cells, n = 3. (**D**) Ca^2+^ flux induced by TREM2 ligation is prevented in PLCγ2 Ko cells. n = 3 (**E**) PLCγ2 deficiency abolishes TREM2-induced IP1 production as determined by HTRF assay. n = 3 (**F**) Representative Western blots and (**G**) quantification of ERK1/2 phosphorylation shows no major difference between Parent and PLCγ2 Ko cells. Full immunoblot images are presented in Supplementary Fig. 10. Data shown represent mean ± SEM, two-way ANOVA followed by Bonferroni’s multiple comparison test, *p < 0.05, **p < 0.01, ***p < 0.001.

We next assessed TREM2 signalling in the PLCγ2 Ko iPSC-macrophages following stimulation with the anti-TREM2 polyclonal antibody AF1828 and observed a similar degree of SYK phosphorylation by Western blot as seen in the Parent line (Fig. 3A, B), indicating that signalling upstream of PLCγ2 was not affected, at least when a strong activator such as the polyclonal antibody is applied. We confirmed SYK phosphorylation using HTRF technology, a sensitive assay that enables the detection of small changes more reliably than Western blot. Again, SYK phosphorylation in PLCγ2 Ko cells was not significantly reduced upon stimulation with AF1828 (Fig. 3C). Although SYK phosphorylation upstream of PLCγ2 was normal, the calcium response upon TREM2 ligation was completely absent in PLCγ2 Ko macrophages, indicating that the TREM2-induced Ca^2+^ flux is exclusively mediated by PLCγ2 (Fig. 3D). The lack of Ca^2+^ signal was confirmed in all three Ko clones available (Suppl. Figure 3C). Despite their lack of response upon TREM2 ligation, we confirmed normal responses to ionomycin and ATP (Suppl. Figure 3E, F). IP1 production occurring in Parent cells upon TREM2 ligation is likewise prevented in PLCγ2 Ko cells (Fig. 3E), confirming the dependency of inositol signalling downstream of TREM2 on PLCγ2 and indicating the lack of a role for the constitutive PLCγ1. We also assessed the effect of PLCγ2 Ko on the activation of MAPKs such as ERK1/2. TREM2 ligation lead to phosphorylation of ERK1/2, which was not significantly different between Parent and PLCγ2 Ko, indicating no downstream effect of PLCγ2 on activation of these MAPKs (Fig. 3F, G).

### PLCγ2 Ko iPSC-macrophages display deficits in survival and phagocytosis, but not LPS-induced TNFα and IL-6 secretion

**Figure 4:**
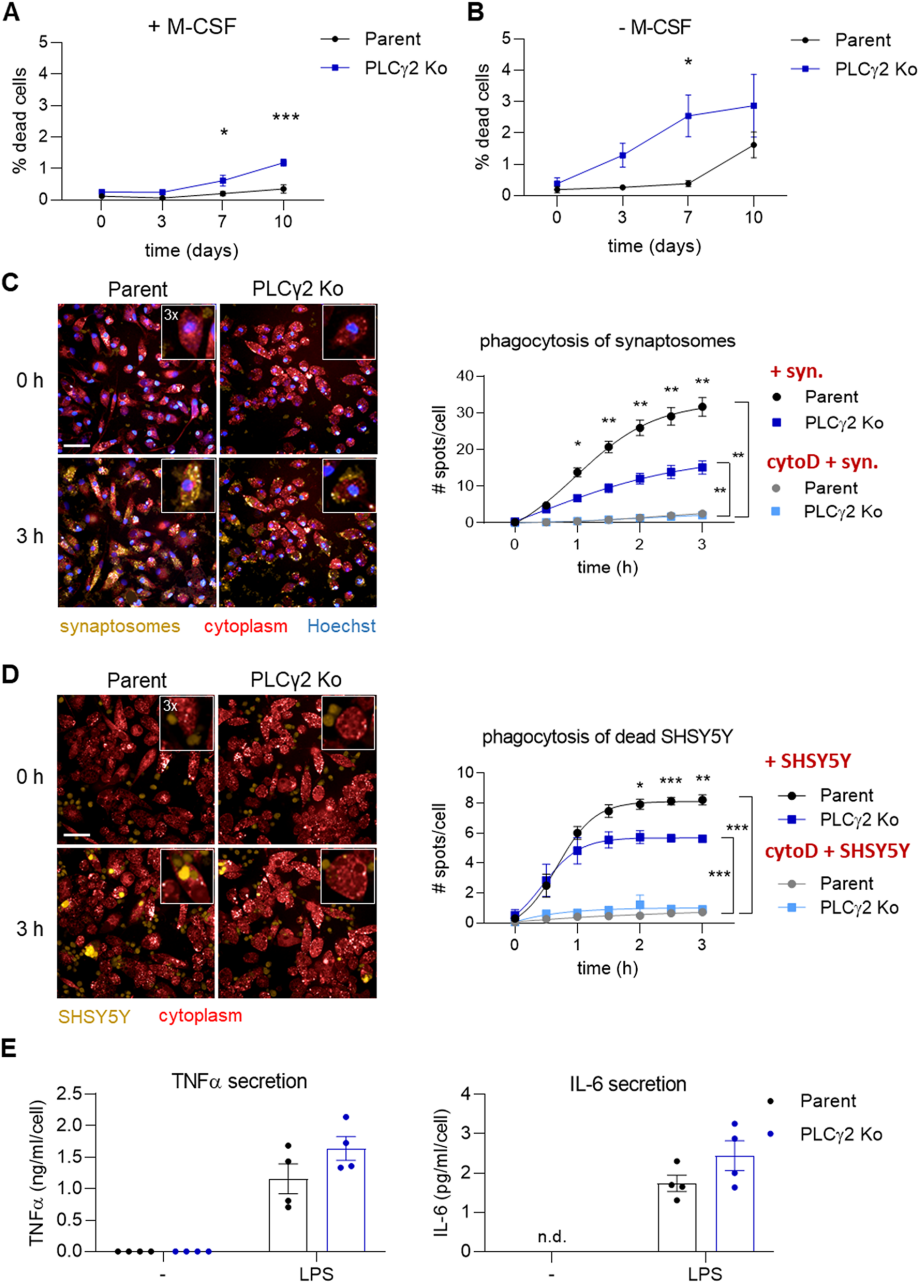
Survival and phagocytosis are reduced in PLCγ2 Ko iPSC macrophages, while LPS-induced secretion of inflammatory cytokines TNFα and IL-6 is unaffected. (**A**) Cell death is slightly increased under normal culture conditions after ≥ 7 days in the PLCγ2 Ko line compared to the Parent. n = 3 (**B**) In the absence of M-CSF, PLCγ2 Ko cells show enhanced sensitivity to cell death, as the percentage of dead cells is increasing earlier than in the Parent line. n = 3 (**C**) Representative live-cell images of human iPSC macrophages phagocytosing pHrodo-labelled synaptosomes and quantification of phagocytic uptake indicated as number of spots per cell. PLCγ2 Ko cells show decreased phagocytosis rate compared to Parent. Cytochalasin D (cytoD, 10 µM) pre-incubation prevents phagocytic uptake in both lines. n = 4. Scale bar 50 μm, inset is a section of the image magnified threefold. (**D)** Representative live-cell images of macrophages phagocytosing pHrodo-labelled dead SHSY5Y and quantification of phagocytic uptake indicated as number of spots per cell. PLCγ2 Ko cells show decreased phagocytosis rate compared to Parent. Cytochalasin D pre-incubation prevents phagocytic uptake in both lines. n = 4. Scale bar 50 μm, inset is a section of the image magnified threefold. (**E)** Secretion of TNFα and IL-6 after 24 h stimulation with LPS is not affected in the absence of PLCγ2, n = 4. n.d. – not detectable. Data shown represent mean ± SEM, two-way ANOVA followed by Bonferroni’s multiple comparison test. * p < 0.05, ** p < 0.01, *** p < 0.001.

We next sought to investigate functional effects of PLCγ2 Ko on iPSC-macrophage phenotype. Previously, a TREM2-dependent survival defect has been shown in TREM2 Ko iPSC-derived microglia-like cells^23,26^, a deficit that is also present in cells lacking PLCγ2^26^. Therefore, we aimed to validate this phenotype in the PLCγ2 Ko iPSC-macrophages. In the absence of M-CSF, an important macrophage growth and differentiation factor that is known to drive survival and proliferation via CSF1R, PLCγ2 Ko cells showed a reduced survival rate, determined by a slightly higher degree of dead cells in the culture than Parent cells (Fig. 4B, Suppl. Figure 4), confirming its importance in regulating survival mechanisms in macrophages. Interestingly, PLCγ2 Ko cells also showed a slight but significant deficit in survival in the presence of M-CSF (Fig. 4A) that was not previously observed in TREM2 Ko cells^23^, indicating that perhaps CSF1R-mediated pro-survival pathways also incorporate PLCγ2 as a signalling component.

Phagocytic activity of iPSC-derived microglia-like cells has recently been shown to be reduced by PLCγ2 deficiency^26^, whilst phagocytosis was enhanced in bone marrow-derived macrophages that express the hypermorphic P522R variant^19^. To determine the effect of PLCγ2 Ko on phagocytic capacity in our iPSC-macrophage model, we used pHrodo-labelled rat synaptosomes or dead SHSY5Y as phagocytic cargo. Both substrates expose surface phosphatidylserine and were recently shown to be taken up by iPSC-derived macrophages at least partially via TREM2-dependent mechanisms^23^. Using live-cell high-content imaging, we observed a steady uptake of synaptosomes and SHSY5Y over the course of three hours, a process that was almost completely abolished by actin inhibitor cytochalasin D (Fig. 4C, D). Notably, PLCγ2 Ko cells phagocytosed both cargos to a lesser degree than Parent cells, indicating that PLCγ2 is involved in transferring signals that facilitate phagocytic activity, possibly downstream of TREM2.

In addition to mediating TREM2-dependent signalling, PLCγ2 has been implicated in driving inflammatory responses in mouse BMDMs and in iPSC microglia-like cells, via TLR and inflammasome activation^19,26^. Challenging the iPSC-derived macrophages with *E.coli* LPS induced the secretion of TNFα and IL-6, but did not reveal any differences in release of those cytokines in PLCγ2-deficient cells (Fig. 4E).

### Adhesion and spreading on substrates of the extracellular matrix is impaired in PLCγ2 Ko cells

**Figure 5:**
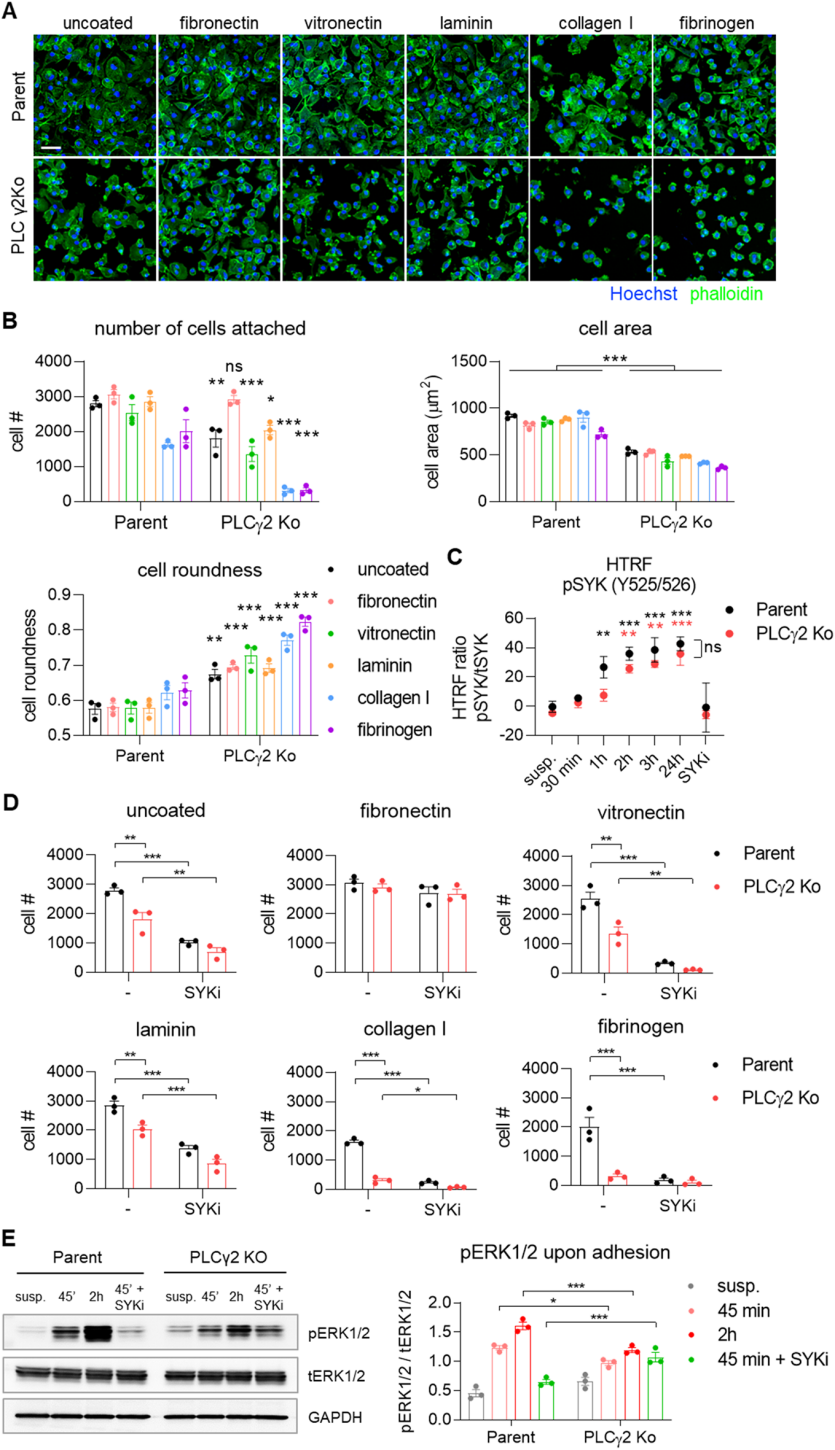
Cell adhesion to different substrates is compromised in PLCγ2 Ko macrophages. (**A**) Representative images of Parent and PLCγ2 Ko cells attached to surfaces coated with the ECM molecules indicated. Scale bar 50 μm (**B**) Quantification of number of cells attached, cell area and cell roundness 3 h after plating on ECM molecules. n = 3; annotations compare Parent vs. PLCγ2 Ko for each coating. (**C**) Phosphorylation of SYK is increased after different time points upon adhesion, as opposed to cells kept in suspension (susp.) in Parent and PLCγ2 Ko line, n = 3; black annotations compare Parent adhered vs. suspension, red annotations compare PLCγ2 Ko adhered vs. suspension (**D**) Effect of SYK inhibition using BIIB-057 (SYKi) on cell adhesion, demonstrating SYK-dependency of adherence to uncoated plates, vitronectin, collagen I, laminin and fibrinogen, but not to fibronectin. n = 3 (**E**) Representative Western blot showing that phosphorylation of ERK1/2 upon adhesion is decreased in PLCγ2 Ko cells. Full immunoblot images are presented in Supplementary Fig. 11. n = 3, data shown represent mean ± SEM, two-way ANOVA followed by Bonferroni’s multiple comparison test. *p < 0.05, **p < 0.01, ***p < 0.001.

Previous reports demonstrating the involvement of SYK-dependent signalling pathways downstream of integrin in neutrophils and macrophages^32^ and specifically the role of PLCγ2 in integrin-mediated neutrophil functions^33^ prompted us to presume a similar role of PLCγ2 during integrin-mediated cell adhesion and spreading in iPSC-derived macrophages. Macrophage adhesion and spreading is regulated by several integrin receptors including β1, β2 and β3 integrins, which transduce signals inside the cell to regulate rearrangement of the actin cytoskeleton, cell movement, and to integrate these signals with those of other transmembrane receptors to coordinate anchorage-dependent cellular functions^34^. We aimed to determine whether the absence of PLCγ2 would impair the functions of adhesion receptors. Therefore we plated fully differentiated Parent or PLCγ2 Ko iPSC-macrophages on uncoated plates (TC-treated plastic) or plates coated with different extracellular matrix (ECM) substrates, namely fibronectin, vitronectin, laminin, collagen type I or fibrinogen, which facilitate adhesion via different integrin receptors, and allowed adhesion for 3 h. Parent cells were able to attach and spread on all coatings tested, with a slightly lower degree of adhesion to collagen type I and fibrinogen. We quantified the cell area and cell roundness as a measure of cell spreading, based on a phalloidin staining to visualize the actin cytoskeleton, and observed that cell spreading in Parent cells was not affected by coating with different ECMs (Fig. 5A, B). In contrast, PLCγ2 Ko cells showed impaired ability to attach and spread on uncoated plastic, indicated by a lower degree of adherent cells, as well as a smaller cell area and a higher degree of cell roundness (Fig. 5A, B). Adhesion and spreading of Ko cells on vitronectin and laminin were likewise impaired, while the deficits in attachment were exacerbated further by collagen type I and fibrinogen, with few cells being able to attach and weak spreading on these substrates (Fig. 5A, B). These data illustrate the severe deficits in cell attachment and spreading of the PLCγ2 Ko iPSC-macrophages with respect to several ECM molecules, indicating disturbances in a number of integrin receptor systems. An exception was fibronectin, which reversed the adhesion phenotype of PLCγ2 Ko cells, demonstrating similar degree of cell adhesion as the Parent iPSC-macrophages. Even though adhesion was rescued, fibronectin coating did not rescue the cell spreading phenotype of the Ko cells, since cell area and roundness were not affected (Suppl. Figure 5). This suggests that initial adhesion to fibronectin is facilitated by PLCγ2-independent mechanisms, while spreading on fibronectin is dependent on PLCγ2.

It is known that integrin-mediated activation of monocytes requires SYK^35^, so we next aimed to investigate whether adhesion of the iPSC-derived macrophages activates SYK-mediated signalling. Therefore we analysed SYK phosphorylation in cells that were allowed to adhere for different amounts of time, as opposed to cells that were kept in suspension. In Parent iPSC-macrophages, cell adhesion led to increased levels of phosphorylated SYK compared to cells that were non-adherent from 1 h onwards, indicating activation of integrin signalling in macrophages upon attachment (Fig. 5C). The presence of a SYK inhibitor prevented adhesion-dependent phosphorylation of SYK. In PLCγ2 Ko cells, SYK phosphorylation was slightly delayed, with a significant increase of phospho-SYK observed from 2 h after plating. At later time points SYK phosphorylation was not significantly affected by the absence of PLCγ2, confirming functional upstream signalling (Fig. 5C).

Since we detected SYK activation upon adhesion, we next investigated whether signalling via SYK is required to facilitate cell attachment of iPSC- macrophages to different molecules of the ECM. To address this we used BIIB-057 to prevent SYK signalling during adhesion and analysed how many cells attached during a time frame of 3 h (Fig. 5D). Inhibition of SYK reduced adhesion of Parent cells to uncoated plates and all ECM substrates apart from fibronectin, indicating that the adherence of iPSC- macrophages to most of the ECM components tested is dependent on functional signalling via SYK. In PLCγ2 Ko cells the deficits in cell adhesion were further exacerbated in the presence of the SYK inhibitor, apart from adherence to fibrinogen, which was nearly prevented at baseline and could not be further reduced. The additional effect of the SYK inhibitor suggests upstream regulation of PLCγ2 by SYK, and potentially a partial compensation of PLCγ2 function by other mechanisms. Again, an exception was attachment to fibronectin which was not affected by SYK inhibition in either Parent or Ko iPSC-macrophages, suggesting mechanisms independent of SYK and PLCγ2 signalling facilitating adhesion. Although cell adhesion to fibronectin was not affected by SYK inhibition, there was a significant increase in cell roundness in the Parent line, indicating that SYK is partially involved in regulating cell spreading on fibronectin (Suppl. Figure 5).

Another indicator of integrin-induced macrophage activation that has been previously described is the analysis of ERK phosphorylation induced upon adhesion, which requires both the CD18 integrin chain and SYK^32^. We hypothesised that PLCγ2 Ko would disrupt ERK signalling. Adhesion to uncoated surface caused an increase in phospho-ERK1/2 after 45 min in Parent cells, which further increased after 2 h of attachment (Fig. 5E). This response was entirely prevented in the presence of the SYK inhibitor, indicating SYK-dependency of ERK1/2 activation. In contrast, ERK1/2 phosphorylation was weakened in PLCγ2 Ko cells, indicating that PLCγ2 is partially required to facilitate phosphorylation of ERK1/2. The residual ERK response to adhesion observed in PLCγ2 Ko cells was SYK-independent, suggesting the occurrence of compensatory mechanisms in the absence of PLCγ2.

**Figure 6:**
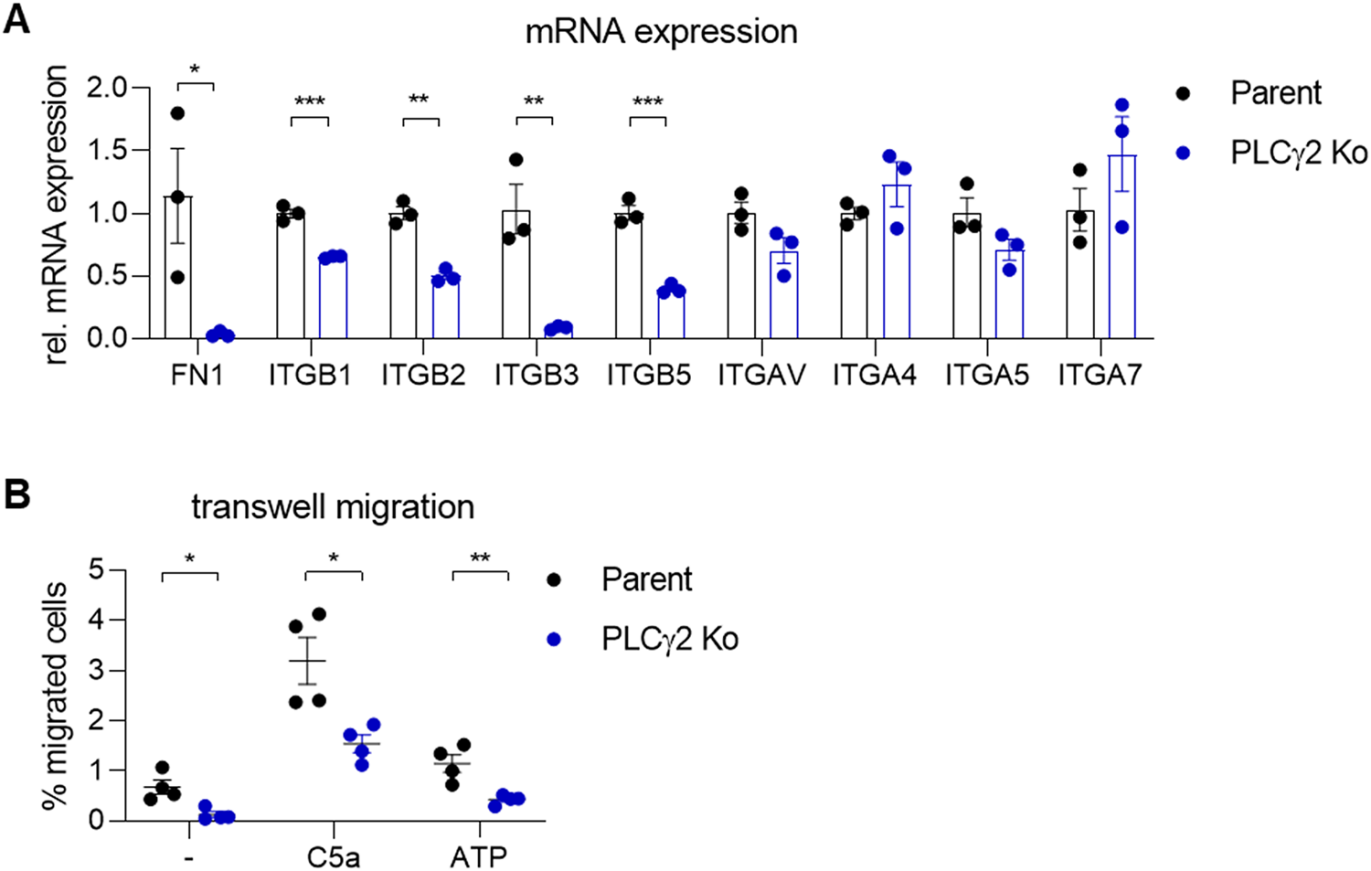
Dysregulation of integrin subunits and deficits in migratory behaviour in PLCγ2 Ko macrophages. (**A**) RT-PCR analysis shows a reduction of fibronectin (FN1) and several β-subunits in PLCγ2 Ko cells. n = 3, (**B**) Migration without chemotactic cue and directed migration towards C5a (3 nM) and ATP (1 mM) are reduced in PLCγ2 Ko cells, n = 4. Data shown represent mean ± SEM, Multiple Student’s t-tests. *p < 0.05, **p < 0.01, ***p < 0.001.

In order to determine whether intrinsic differences in integrin expression in PLCγ2-deficient cells might contribute to the observed phenotypic deficits in cell adhesion, we measured the expression of several integrin subunits known to be expressed by macrophages using qRT-PCR. Strikingly, gene expression of all the beta-integrin subunits analysed (ITGB1, ITGB2, ITGB3 and ITGB5) were significantly reduced in PLCγ2 Ko iPSC-macrophages, while expression of alpha-integrin subunits (ITGAV, ITGA4, ITGA5 and ITGA7) were unaltered (Fig. 6A). Additionally, fibronectin (FN1) was downregulated in PLCγ2 Ko cells, which could result in reduced fibronectin deposition and thus, could explain why coating surface with fibronectin enhances macrophage attachment in the Ko. Overall, this demonstrates profound disturbances in several integrin receptor systems in the absence of PLCγ2, which provides an explanation for the pronounced adhesion deficits to several ECM molecules we observed.

### PLCγ2 Ko leads to impairment of macrophage motility and directed migration

The ability to form proper cell adhesion is necessary to enable motility and directed cell movements. Given the pronounced cell adhesion deficits in PLCγ2 Ko iPSC-macrophages, we hypothesised that cell motility and chemotaxis were likewise affected. To investigate this we monitored cell migration using transwell inserts, in the absence or in the presence of a chemotactic cue. We observed a clear deficit in non-directional motility in the Ko, as the cells remained static and did not show any movement in the absence of a stimulus (Fig. 6B). Adding a chemotactic stimulus like C5a or ATP to the bottom of the well induced some directed migration of the PLCγ2 Ko cells, although to a significantly lower degree than the Parent cells. We conclude that in addition to deficits in cell adhesion, PLCγ2 Ko also causes an impairment in cell motility and migration.

## Discussion

The previous discovery of AD-related mutations in the microglial expressed genes TREM2 and PLCγ2 has marked them as promising new targets to manipulate microglial function in neurodegenerative disease. While several variants in TREM2 associated with loss of function were found to increase AD risk^2,36,37^, a mutation in PLCγ2 with a hypermorphic effect on enzymatic activity has been shown to be protective^18,19^, indicating that boosting TREM2 and/or PLCγ2 function could be beneficial in AD.

Recently, the link between TREM2 and PLCγ2 in human microglia has been established, placing PLCγ2 downstream of TREM2 signalling, and suggesting it as a key node to regulate TREM2 functions but also inflammatory signalling via TLRs^26^. In this study, we confirmed some of those previous findings and extended them by demonstrating additional functional effects of PLCγ2 deficiency on myeloid cell phenotype using iPSC-derived tissue-type macrophages. Stimulating TREM2 signalling using a TREM2-specific antibody, we observed PLCγ2 enzymatic activity, evidenced by Ca^2+^ flux and IP1 production. These cellular responses upon TREM2 ligation were completely absent in iPSC-macrophages deficient in PLCγ2, suggesting that calcium release downstream of TREM2 is exclusively triggered by PLCγ2 in human iPSC-macrophages. This observation adds to previous reports showing that PLCγ2 is similarly required in other peripheral immune cells to enable calcium mobilization upon B cell receptor activation using anti-IgM^38^, as well as FcεR ligation by IgE in mast cells and FcγR activation in macrophages^10^. Thus, we observed a similar non-redundant role of PLCγ2 in the context of TREM2 signal transduction. We further confirmed that TREM2-induced PLCγ2 activity requires recruitment and activation of SYK, as inhibition of SYK activation prevented the PLCγ2-induced calcium signal. Our data complement the recent report showing similar effects on PLCγ2 activity using liposomes as a TREM2 stimulant, demonstrating that PLCγ2 is activated downstream of the activated TREM2-DAP12-SYK complex^26^.

PLCγ2 proved to be vital for the regulation of several macrophage functions, e.g. promoting survival, phagocytosis, cell adhesion and migration. Some of these functions have been previously shown to involve TREM2 such as survival and phagocytosis^23,26,39–41^, whilst others could be less dependent on TREM2 and suggest a role of PLCγ2 downstream of other receptor complexes. Phagocytosis of cortical synaptosomes and dead cells is initiated by detection of phosphatidylserine on their surface, a process that is at least partially mediated by TREM2. Similarly to TREM2 Ko macrophages^23^, genetic deficiency of PLCγ2 significantly reduced phagocytosis of both cargos, indicating that PLCγ2 is required to mediate TREM2-induced effects on phagocytosis. However, we cannot exclude the possibility that the reduction in phagocytic activity of PLCγ2 Ko iPSC-macrophages relative to wildtype cells is a direct result of their reduced TREM2 surface expression, which could reduce cargo detection and phagocytic uptake. Although we did not observe an impairment of signalling upstream of PLCγ2 using TREM2-activating antibody as a TREM2 agonist, the TREM2 antibody is a high-affinity ligand and may saturate TREM2 downstream signalling even when receptor numbers are reduced. In contrast, phagocytic cargo such as dead neurons and synaptosomes are low-affinity ligands of TREM2^24^. Moreover, the finding that several integrin receptor systems are downregulated upon PLCγ2 deficiency could also be directly linked to deficits in phagocytosis, as the process of phagocytosis crucially relies on integrin-dependent remodelling of the actin cytoskeleton^42^. In concordance with our data, Andreone et al. found similar functional defects of PLCγ2 Ko, such as reduced survival and phagocytosis of myelin debris^26^. In contrast, mouse BMDMs expressing the P522R mutation in PLCγ2 display enhanced phagocytosis, indicating that the hypermorphic variant affects the TREM2-PLCγ2 pathway in the opposite direction^19^.

There are various reports on the role of PLCγ2 in regulating the inflammatory response. PLCγ2 has been implicated in NLRP3 inflammasome activation evidenced by IL-1β production in peripheral immune cells^43^ and in iPSC-derived microglia cells^26^. It was also shown that genetic deficiency of PLCγ2 lead to reduced cytokine secretion upon TLR2 stimulation using zymosan^26^. The hypermorphic variant on the other hand increased secretion of inflammatory cytokines upon acute stimulation with LPS and IFNγ^19^. In PLCγ2 Ko macrophages, we did not observe differences in secretion of inflammatory cytokines TNFα and IL-6 when activating the TLR4 pathway with LPS. The effect of this specific stimulation paradigm has not yet been explored in PLCγ2-deficient cells, and our results indicate that PLCγ2 is not directly involved in inflammatory cytokine secretion downstream the TLR4-MyD88-NFκB pathway, specifically of TNFα and IL-6. However it might have other effects on TLR4 signalling. PLCγ2 has been previously associated with LPS-induced TLR4 endocytosis and subsequent IRF3-mediated signalling from the endosome, leading to IFNα/β production, but it was shown not to be involved in MyD88-dependent TLR4 signalling and TNFα secretion^44,45^. As such, our results are in line with the observation that LPS-induced secretion of TNFα and other inflammatory cytokines is not affected in the absence of PLCγ2.

We observed that deletion of PLCγ2 impaired cell adhesion to several ECM molecules, and furthermore impaired cell spreading and migration, and that those deficits corresponded with reduced expression of several integrin receptor subunits. Thus, our study provides important evidence that the genetic deficiency of PLCγ2 majorly compromises integrin-mediated adhesion in human macrophages. In our cell model, this phenotype is probably due to a variety of intrinsic changes inherent in macrophages in which PLCγ2 is depleted, such as decreased expression of integrin receptors and possibly a number of other receptor systems. However, previous report have demonstrated a direct involvement of PLCγ2 in signalling pathways downstream of integrin receptors. In neutrophils, PLCγ2 was implicated in mediating intracellular mobilization and influx of Ca^2+^ upon engagement of β2 integrin^46^. It was shown that PLCγ2 was required to facilitate adhesion-dependent neutrophil activation and spreading upon integrin ligation^33^. Integrin signalling in macrophages uses similar pathway components as neutrophils, including the ITAM-containing adaptor proteins DAP12 and FcRγ, which activate SYK downstream of β2 integrin^32^. It is likely that PLCγ2 similarly participates in signalling cascades downstream of integrin in human macrophages. However, the severe phenotype we observed in our cell model is most likely due to the transcriptional downregulation of the β integrin receptor subunits, perhaps in combination with defective integrin downstream signalling due to the lack of PLCγ2.

Alongside deficits in cell adhesion, we also observed dysfunctional migration and chemotaxis in PLCγ2 Ko iPSC-macrophages. Cell-substrate adhesions affect migration speed, with low or high adhesive strength slowing down migration, while intermediate adhesive strength enables optimal migration^47^. It is thus likely that the inability to form normal integrin-mediated adhesions in the Ko would hamper migratory behaviour. Indeed, we observed reduced baseline motility as well as reduced directed migration using two different chemotactic cues. Apart from an indirect effect on migration by regulating integrin expression and adhesion, there is a possibility that PLCγ2 directly influences migration by contributing to Ca^2+^ signalling during migration. Local changes in intracellular calcium play a critical role in regulating cell migration and chemotaxis in diverse cell types^48–50^. Recruitment of PLCγ2 to the membrane, a well-characterized mechanism of PLC activation, has been observed during chemotaxis of neutrophils, with PLCγ2 being localised to the leading edge of migrating cells^51^. PLCγ2 was involved in signalling downstream of Bruton’s tyrosine kinase (Btk) leading to Mac-1 activation and neutrophil recruitment in a mouse model of sterile inflammation^52^. A role of PLCγ2 has also been shown during B cell migration, where it was involved in chemokine-induced migration downstream of Btk^53^. A similar role of PLCγ2 mediating Ca^2+^ signals during chemotaxis is also feasible in macrophages, and requires further mechanistic investigation.

The vital role of PLCγ2 in multiple myeloid cell functions is highlighted by the severe dysfunction of phagocytosis, survival, cell adhesion and migration in PLCγ2-deficient iPSC-macrophages. As an integral component of signalling pathways downstream of several immune receptors, including TREM2, a loss of function in PLCγ2 affects a variety of cellular processes. PLCγ2 deficiency resulted in pronounced changes in expression of macrophage receptors like several integrin subunits and TREM2, which could provide the molecular basis for the observed functional effects, or at least contribute to the severity of the phenotypic deficits. Further research is needed to determine the precise mechanisms by which PLCγ2 impacts integrin and ECM expression and how this could be implicated in AD pathogenesis. The discovery of the protective AD variant P522R, which slightly increases enzymatic activity, suggests that enhancing PLCγ2 activity could be a viable strategy to increase beneficial microglial functions in the context of AD.

## Methods

### Generation of PLCG2 KO iPSC lines and culturing of human iPSC-derived macrophages

iPSC lines BIONi010-C (Parent, Bio Sample ID: SAMEA3158050, ECACC ID: 66,540,023) and BIONi010-C-17 (TREM2 KO, Bio- Sample ID: SAMEA104386270, ECACC ID: 66,540,632) were obtained from Bioneer and are available from the European Collection of Authenticated Cell Cultures (ECACC). PLCγ2 KO iPSC lines were generated by Bioneer in the BIONi010-C parent iPSC line using CRISPR-Cas9 technology. Two different modified sgRNA’s targeting Exon 1 and Intron 1 of *PLCG2* or 1 modified sgRNA targeting Exon 1 of *PLCG2* and a single stranded oligodeoxynucleotide (ssODN) encoding a STOP codon and a XbaI restriction site was co-delivered with Hi-Fi CRISPR-Cas9 protein as a ribonucleoprotein complex. Both strategies resulted in a knockout in the first coding exon that is present in all isoforms of the *PLCG2* gene, leading to a premature stop of translation. The BIONi010-C PLCγ2 KO clones 20, 53 and 93 were selected and further characterised. Large-scale SNP quality-controlled batches were frozen at p15–25 and used for experiments within a minimal number of passages post-thaw to ensure consistency. An Illumina Omniexpress 24 v1.2 SNP microarray analysis was performed to verify genomic integrity, as previously described in Haenseler et al.^29^.

iPSC were grown on hESC-qualified Geltrex-coated plates (Gibco) in mTeSR™1 media (STEMCELL Technologies) and passaged as clumps using 0.5 mM EDTA in PBS. iPSC were differentiated to primitive, tissue-type macrophages as previously described^28^. In brief, iPSC were seeded into Aggrewell-800 wells (STEMCELL Technologies) to form embryoid bodies and fed daily with mTeSR™1 media supplemented with 50 ng/mL BMP4 (Peprotech), 50 ng/mL VEGF (Peprotech) and 20 ng/mL SCF (Miltenyi Biotec). In a modification to the previously published protocol, the embryoid bodies were cultured for 5 days in growth factors instead of 4 days, and after the first 2 days they were transferred into low-adherence 6-well plates. Embryoid bodies were then transferred to T175 flasks, known as ‘differentiation factories’, and fed weekly with X-VIVO15 (Lonza) containing 100 ng/mL M-CSF, 25 ng/ml IL-3, 2 mM Glutamax, 2-mercaptoethanol, 100 U/mL penicillin and 100 μg/mL streptomycin (all Life technologies). iPSC-macrophage precursors, emerging into the medium after approximately 2–3 weeks, were harvested weekly, plated in their final assay format and differentiated to iPSC-macrophages for 6–9 days at 37 °C and 5% CO_2_, in X-VIVO15 with 100 ng/mL M-CSF, 2 mM Glutamax, 100 U/mL penicillin and 100 μg/mL streptomycin (macrophage media). Cells received one 50% medium change on day 2 or 3.

### Flow cytometry for macrophage surface markers

Macrophage precursors were plated at 1 × 10^6^ cells/well in 6-well plates and differentiated in macrophage medium for a week. Macrophages were lifted from 6-well plates by incubation with StemPro Accutase (Gibco) for 10 min at 37 °C. The cells were washed with PBS and non-specific binding sites were blocked by incubation in FACS buffer (PBS, 1% FCS, 10 μg/mL human IgG) for 10 min at RT. 2 × 10^5^ cells per sample were stained with directly-conjugated primary antibodies against CD11b (clone ICRF44, Biolegend), CD14 (clone 18D11, Immunotools) and CD45 (clone MEM-28, Immunotools), for 30 min at RT. Cells were then washed twice with FACS buffer and fixed with 4% paraformaldehyde (PFA) for 10 min at RT. Cells were washed with PBS and analysed on a FACS Calibur flow cytometer (BD Biosciences). Fluorophore-conjugated isotype controls from the same manufacturers were used.

### RNAScope

Macrophage precursors were seeded at 3 × 10^4^ cell/well in optically-clear bottom CellCarrier 96-well plates (Perkin Elmer), and differentiated in macrophage media for a week. Cells were fixed with 4% PFA, and In situ hybridization (ISH, RNAScope Multiplex Fluorescent v2 323,110, ACD Biotechne) was carried out according to manufacturer’s instructions with catalogue probe Hs-PLCG1 472,801. ISH was followed by immunocytochemistry with anti-Iba1 (1:500, Q08578, Alpha Laboratories). Images were taken on a high content Imaging system (Operetta, Perkin Elmer) and analysed using Harmony Software (Perkin Elmer) to quantify the number of dots (RNASCope reaction products) per cell.

### SDS-PAGE and Western blot

Macrophage precursors were plated at 1 × 10^6^ cells/well in 6-well plates and differentiated in macrophage medium for a week. To induce TREM2 signalling, cells were stimulated with a goat polyclonal human TREM2 antibody (AF1828, R&D systems) at 1, 2, 5 or 10 µg/ml for 5 min at 37 °C. Normal goat IgG (R&D Systems) was used as control. For experiments analysing ERK1/2 phosphorylation upon cell adhesion, cells were lifted with StemPro Accutase (Gibco) for 10 min at 37 °C, washed with PBS and either kept in suspension or re-plated into 6-well plates for 45 min or 2 h, in the absence or presence of the SYK inhibitor BIIB-057 at 5 μM (Cambridge Bioscience). To harvest cells, medium was aspirated and cells were lysed with 120 μl Pierce IP Lysis Buffer (Thermo Scientific), supplemented with protease and phosphatase inhibitor cocktail (Sigma, Roche). Homogenates were centrifuged at 14,000 xg and the supernatants were collected. Protein content was quantified using Pierce Coomassie (Bradford) Protein Assay Kit (Thermo Scientific), following manufacturer’s instructions.

30 μg of protein cell lysates were loaded into Novex 8–16% Tris–Glycine precast midi gels (Thermo Scientific) and transferred to a nitrocellulose membrane using the Trans-Blot® Turbo™ RTA Mini Transfer Kit (BioRad). After blocking with 5% BSA in TBS/0.1% Tween20, membranes were incubated with primary antibodies diluted in blocking buffer over night at 4 °C . Antibodies were purchased from commercial sources and are listed in Table 1. Membranes were washed in TBS/0.1% Tween20 and further incubated with an HRP-labelled anti-rabbit IgG or anti-mouse IgG (Thermo Scientific) for 1 h at RT. Membranes were incubated with the SuperSignal West Dura Extended Duration Substrate (Thermo Scientific) and signal was detected on the FujiFilm LAS-4000 System (Raytek). Intensity of protein bands was quantified using ImageJ 1.52a software.

**Table 1:** Primary antibodies used for Western blot.

Antigen	Host	Supplier	Catalog number	Dilution
Phospho-SYK (Y525/Y526)	rabbit	Thermo Scientific	MA514918	1:500
Total SYK	mouse	Genetex	GTX31122	1:1000
Total PLCγ2	rabbit	CST	3872S	1:500
Phospho-p44/42 MAPK (T202/Y204)	rabbit	CST	4370S	1:1000
Total p44/42 MAPK	rabbit	CST	4695S	1:1000
TREM2	rabbit	Abcam	ab209814	1:500
GAPDH	rabbit	Sigma	G9545	1:2000

### Calcium assay

Macrophage precursors were seeded at 1 × 10^4^ cells/well in optically-clear bottom CellCarrier 384-well plates (Perkin Elmer) and differentiated in macrophage medium for 7 days. A 384-well source plate containing 5 × concentrated stimuli ATP (final concentration 0.5 mM, Sigma) or TREM2 antibody AF1828 (final concentration 1, 2, 5 or 10 μg/mL, R&D Systems) was prepared for transfer onto the macrophages. Macrophages were loaded with 25 μL of 4 μM calcium-sensitive dye Fluo4-AM (Thermo Scientific) in the presence of 0.05% pluronic acid (Life technologies) diluted in HBTS buffer (HEPES Buffered Tyrode’s Solution: NaCl 135 mM, KCl 5 mM, MgCl_2_ 1.2 mM. CaCl_2_ 2.5 mM, HEPES 10 mM, glucose 11 mM, pH 7.2) for 1 h at RT. To determine the effect of SYK inhibition on the TREM2-evoked Ca^2+^ signal, the SYK inhibitor BIIB-057 (Cambridge Bioscience) was added to the Fluo4-AM solution at indicated concentrations and incubated for 1 h simultaneously with the Ca^2+^ dye. Macrophages were washed with HBTS and loaded onto the FLIPR Tetra system (Molecular Devices). Using the pipettor function, 10 µl of stimuli from the source plate were pipetted onto the plate containing the cells in 40 µl buffer/well, achieving a 1:5 dilution of the stimuli. Each condition was run in quadruplicate. Relative fluorescent units (RFU) of the assay plate were read with the excitation/emission pairs 470–495 nm LEDs and 515–575 nm emission filters. Settings were adjusted in order to have values of ~ 1000 RFUs at baseline. Basal fluorescence was measured for 1 min and following injection of stimuli, the response was recorded for 5 min at reading intervals of 1 s using the ScreenWorks software. Data was exported as maximum-minimum signal or calculated area under the curve and RFU normalised to baseline values set to 100%.

### IP1 detection by HTRF assay

iPSC macrophage precursors were plated into optically-clear bottom CellCarrier 384-well plates (Perkin Elmer) at a density of 5 × 10^4^ cells/well and differentiated for 7 days in macrophage medium. For detection of IP1, the IP-ONE—Gq Kit (62IPAPEC, Cisbio) was used, according to manufacturer’s instructions. In brief, medium was removed and 20 µl of stimulation buffer (StimB) was added per well. Goat polyclonal TREM2 antibody (R&D Systems, AF1828) or normal goat IgG control (R&D systems, #AB-108-C) were diluted to 3.5 × of final concentration in StimB, and 8 µl were added directly into the wells. Cells were incubated for 2 h at 37 °C. A standard curve was generated according to manufacturer’s protocol and transferred to a white ProxiPlate-384 Plus microplate (Perkin Elmer). HTRF pair IP1-d2 and anti-IP1-Cryptate were diluted in Lysis & Detection buffer (Cisbio) and added to each 96 well, as well as to the standards in the 384 well plate. Cells were lysed for 1 h at RT, then half of the cell lysate was transferred to the ProxiPlate-384 Plus. The plate was read on the PHERAstar FSX (BMG Labtech) using a HTRF optic to detect emission at 665 nm and 620 nm. The fluorescence ratio (665 nm/620 nm) of acceptor and donor emission signals was calculated, and final IP1 concentrations were interpolated from the standard curve using nonlinear regression.

### pSYK detection by HTRF assay

Macrophage precursors were seeded at 4 × 10^4^ cell/well in optically-clear bottom CellCarrier 96-well plates (Perkin Elmer), and differentiated in macrophage media for a week. For detection of pSYK, the pSYK/tSYK HTRF kits (Cisbio) were used, according to manufacturer’s instructions. Cells were stimulated with goat polyclonal human TREM2 antibody (AF1828, R&D systems) for 5 min at 37 °C. Normal goat IgG (R&D Systems) was used as control. Medium was aspirated and cells were lysed in supplemented lysis buffer (Cisbio), placed on an orbital shaker for 20 min at room temperature. Experiments were run with 3 replicate wells. Cell lysates were dispensed into a ProxiPlate-384 Plus (Perkin-Elmer), followed by pre-mixed Eu^3+^-cryptate and d2 antibody diluted in detection buffer. The plate was sealed and incubated overnight at RT, then read on a PHERAstar FSX (BMG Labtech) using a HTRF optic to detect emission at 665 nm and 620 nm. Data was exported as two RFU values, and signal/noise ratio was calculated according to manufacturer’s instructions.

### sTREM2 AlphaLISA

Macrophage precursors were seeded at 4 × 10^4^ cell/well into CellCarrier-96 Ultra Microplates (Perkin Elmer) and differentiated to mature macrophages for 8 days. Cell supernatants were collected and triplicate wells were pooled for each genotype. 20 mg/mL AlphaLISA acceptor beads (Perkin Elmer) were washed with PBS, and supernatant was removed following centrifugation at 16,000 × g for 15 min. Beads were conjugated to TREM2 capture antibody (Abcam) by addition of 0.1 mg antibody, 1.25 μL of 10% Tween-20 (Sigma), 10 μL of a 400 mM solution of sodium cyanoborohydride (Sigma) in dH2O and made up to 200µL with 130 mM sodium phosphate buffer (pH 8.0) (Sigma). The bead pellet was resuspended and incubated for 24 h in a 37 °C waterbath. Beads were spun at 16,000 × g for 15 min at 4 °C, then resuspended in 200 µL 100 mM Tris–HCl (pH 8.0) 3 times. Following the last centrifugation, acceptor beads were resuspended at 5 mg/mL in 200 µL PBS + 0.05% Proclin-300 (Sigma), spun down and stored at 4 °C. A standard curve was then generated using human recombinant TREM2 (Sino Biologicals). Diluted standards and supernatants were added to a ProxiPlate-384 Plus (Perkin Elmer), followed by TREM2 conjugated acceptor beads (40 µg/mL final assay concentration) and incubated overnight at 4 °C. The plate was equilibrated to RT, then biotinylated TREM2 antibody (R&D Systems) was added to the plate (1 nM final concentration). After 1 h, streptavidin coated donor beads (Perkin Elmer) (30 µg/mL final concentration) were added. After incubation in darkness for 30 min, the plate was read on a PHERAstar FSX (BMG Labtech) with an AlphaLISA optic exciting at 680 nm and reading emission at 615 nm. Data was exported as relative fluorescent units (RFU) into Prism for interpolating sTREM2 concentrations.

### TNFα and IL-6 ELISA

Macrophage precursors were seeded at 4 × 10^4^ cell/well into CellCarrier-96 Ultra Microplates (Perkin Elmer) and differentiated to mature macrophages for a week. Using triplicate wells per condition, cells were treated with 100 ng/ml *E.coli* LPS (Sigma). Cell supernatants were collected after 24 h of stimulation for detection of IL-6 and TNFα by ELISA. TNFα was measured using the Human TNF-alpha DuoSet ELISA (R&D systems), following manufacturer’s protocol. For detection of IL-6, Greiner high-bind 96 well plates (Sigma) were coated with IL-6 antibody (Life technologies, #14–7069-81) overnight at 4 °C. Plates were washed with PBS + 0.05% Tween20 and incubated with blocking buffer (PBS, 0.05% Tween20 and 1% BSA) to block non-specific binding sites. A standard curve was generated using human recombinant IL-6 (Sino Biologicals). Standard and diluted supernatants were incubated for 2 h at RT. After washing, plates were incubated with a biotinylated antibody against IL-6 (Life technologies, #13–7068-81) for 1 h at RT, followed by incubation with HRP-conjugated streptavidin (Thermo Scientific) for 1 h at RT. Plates were washed and incubated with 1-Step Ultra TMB ELISA substrate solution (Thermo Scientific). The reaction was stopped with 2 N H_2_SO_4_ and the chemiluminescent signal was measured on a plate reader at 450 nm. Data from each well was normalised to the average cell count for that condition (pg/mL/cell).

### qRT-PCR

Macrophage precursors were seeded at 1 × 10^6^ cells/well in 6-well plates and differentiated in macrophage media for 7 days. Medium was aspirated, and cells were lysed with 350 μL Buffer RLT (Qiagen) containing 1% (v/v) 2-mercaptoethanol. Lysates were passed through a blunt 20-gauge needle (0.9 mm diameter) fitted to an RNAse-free syringe at least 5 times and the total RNA was then extracted using the RNeasy Mini kit (Qiagen), according to manufacturer’s protocol. RNA samples were eluted in 30 μL of RNase-free water. The quantity and quality of RNA was measured on a Nanodrop (Thermo Scientific). Reverse transcription was performed using a High-Capacity RNA-to-cDNA kit (Applied Biosystems), with 400 ng RNA input per reaction, following the manufacturer’s protocol. RT-PCR was performed using TaqMan probes and TaqMan Gene Expression Mastermix (Applied Biosystems) in a 384-well PCR plate, 2 μL cDNA in a final volume of 6 μL per well, on a Quant- Studio 5 qRT-PCR machine (Applied Biosystems). TaqMan probes were ITGA4 (Hs00168433_m1), ITGA5 (Hs01547673_m1), ITGA7 (Hs01056475_m1), ITGAV (Hs00233808_m1), ITGB1 (Hs01127536_m1), ITGB2 (Hs00164957_m1), ITGB3 (Hs01001469_m1), ITGB5 (Hs00174435_m1), FN1 (Hs01549976_m1) and TBP (Hs00427620_m1). Samples were run in triplicate wells. ΔCt values were calculated using the average Ct for each triplicate: ΔCt was generated by subtraction of the average Ct for reference gene TBP, and then the ΔCt was normalised to the average ΔCt for all Parent samples (by substraction).

### Survival assay

Macrophage precursors were plated at 4 × 10^4^ cells/well in four separate CellCarrier-96 Ultra Microplates (Perkin Elmer), and differentiated in macrophage medium for 1 week. Survival was assessed as previously described^23^. In brief, cells received a full media change to fresh macrophage medium with or without M-CSF (100 ng/ml), with triplicate wells per condition on each plate. One plate was used to assess the cell number at baseline (day 0), while the other plates were incubated at 37 °C/ 5% CO_2_ for a further 3, 7, or 10 days. The 10-day plate received a 50% medium change at day 7. At the end of each incubation, cells were stained with the ReadyProbes Cell Viability Imaging Kit (Invitrogen) for 30 min at 37 °C/5% CO_2_. Nuclei were imaged using the Opera Phenix High Content Screen System (Perkin Elmer) with a 10 × objective and 9 fields per well. Images were quantified with Columbus 2.7 software (Perkin Elmer). Data was presented as percentage of mean number of dead cells/mean number of total cells for each condition.

### Phagocytosis assay

Macrophage precursors were plated at 3 × 10^4^ cells/well into CellCarrier-96 Ultra Microplates (Perkin Elmer), and differentiated in macrophage medium for a week. Phagocytosis was assessed as previously described, using pHrodo-labelled rat synaptosomes or dead SHSY5Ys as phagocytic cargo^23^**.** SHSY5Ys, cultured in T75 flasks with DMEM/F12 media (Gibco) with 10% FBS (Sigma) and penicillin/streptomycin (Invitrogen), were harvested with TrypLE Express (Gibco), washed with PBS (Gibco), centrifuged at 400xg for 5 min, and re-suspended in Live Cell Imaging Solution (LCIS, Invitrogen). PFA was added to a final concentration of 2%, and the cells were fixed for 10 min at RT. The cells were washed with PBS and centrifuged at 1200xg for 7 min. Rat synaptosomes were kindly provided by Dr Hazel Hall-Roberts and were generated as previously described^23^**.** Before phagocytosis, macrophages were stained for 45 min at 37 °C/5% CO_2_ with 1 μM CellTracker Deep Red (Invitrogen) and 1 drop/mL NucBlue Live ReadyProbes Reagent (Invitrogen). Cells were washed with PBS, and then incubated in XVIVO15 without phenol red (Lonza), with or without 10 μM cytochalasin D (Cayman), a phagocytosis inhibitor, for 1 h at 37 °C/5% CO_2_. The phagocytic cargo (synaptosomes or dead SHSY5Ys) was stained with pHrodo iFL Red STP Ester (Invitrogen), using 20 μg of dye per 1 mg synaptosomes, or 12.5 μg of dye per 1 × 10^6^ SHSY5Ys, aiming for a final concentration of 40 μg/mL. pHrodo-labelling was performed for 30 min at RT, protected from light, in a low protein-binding tube. Cargo was washed twice with HBSS, (centrifugation: 3000xg synaptosomes, 1200xg dead SHSY5Ys), and re-suspended in XVIVO15 without phenol red, to a concentration of 200 ng/μL synaptosomes or 1.2 × 10^6^ cells/mL SHSY5Ys, and 50 μL/well was added to the macrophages. Live cell time-lapse imaging was conducted on the Opera Phenix (Perkin Elmer), with the chamber temperature set to 37 °C and CO_2_ levels set to 5%. Phagocytosis was monitored over the course of 3 h, with repeated measurements every 30 min, using a 40 × water objective, 9 fields/well, with triplicate wells per condition. Images were processed and quantified with Columbus 2.7 software (Perkin Elmer). The parameters measured for each field were average number of spots/cell and the sum of the spot areas.

### Cell adhesion assay

Macrophage precursors were seeded at 1 × 10^6^ cell/well in 6-well plates and differentiated for a week in macrophage media. CellCarrier-96 Ultra Microplates (Perkin Elmer) were coated with 0.5 μg/well (1.56 μg/cm^2^) recombinant human fibronectin (Biolegend), truncated vitronectin (Gibco), laminin (Sigma), collagen type I (Millipore) or fibrinogen (Thermo Scientific), diluted in PBS, by incubation at room temperature for 1 h. The coating solution was aspirated, wells washed once with PBS and non-specific binding sites were blocked with a solution of 10 mg/mL heat-denatured bovine serum albumin (BSA) in PBS for 1 h. Wells were washed once with PBS, and 50 μL fresh macrophage medium ± SYK inhibitor (BIIB-057, Cambridge Bioscience) added (5 μM final well concentration). Macrophages dissociated from the 6-well plates by StemPro Accutase (Gibco) were pelleted and re-suspended in macrophage medium, and 50 μL was added to the coated or uncoated wells at a density of 5 × 10^4^ cells/well. Adhesion was performed for 3 h at 37 °C/5% CO_2_, and then the plates were washed once with PBS and cells fixed for 15 min in 4% PFA at RT. Following two PBS washes, the cells were permeabilised with 0.1% Triton X-100 in PBS for 15 min at RT, then washed twice with PBS and stained with Alexa Fluor 488 Phalloidin (Thermo Scientific) and Hoechst33342 (Thermo Scientific) diluted in PBS for 45 min at RT. Cells were washed 3 × with PBS and imaged on the Opera Phenix, 9 fields of view/well, using a 20 × water objective. Cell numbers and morphological parameters based on the F-actin staining (cell roundness and cell area in cm^2^) were analysed using the Columbus 2.7 software (Perkin Elmer).

### Migration assay

Macrophage precursors were plated at 1 × 10^6^ cells/well in 6-well plates and differentiated in macrophage media for a week. Migration assay was performed as previously described^23^, with slight modifications. Cells were dissociated with StemPro Accutase (Gibco) for 10 min at 37 °C and washed with PBS. Cells were re-suspended in macrophage media, and 100 μL containing 5 × 10^4^ cells was pipetted onto each transwell insert (PET with 5 μm pores, Sarstedt) placed over wells of an empty 24-well plate. In total, 600 μL of macrophage media ± stimuli (1 mM ATP (Sigma) or 3 nM human recombinant C5a (Peprotech)) was added beneath the transwells and incubated for 6 h to allow cell migration. After completion of incubation time, medium was carefully removed, transwell inserts were transferred to a fresh 24-well plate and fixed with 4% PFA for 20 min at RT. Cell nuclei were stained with NucBlue® Live ReadyProbes® Reagent (Thermo Scientific) and imaged with an Olympus CKX53 cell culture microscope, using the 10 × objective with DAPI light cube, taking three images per transwell insert. The transwells were then swabbed with a cotton wool bud to remove cells on the top surface, leaving behind only migrated cells, transferred into a plate with fresh PBS, and imaged again with the same settings. Nuclei counting was performed with ImageJ software version 1.52a, and for each transwell, the % migration was calculated: (no. cells in second scan) ÷ (no. cells in first scan) × 100. Treatments were performed in duplicate and duplicate wells were averaged, then normalised to the average % migration for the entire plate, to control for age-dependent differences in cell speed.

### Statistical analysis

Data are shown as mean ± SEM and were analysed using the GraphPad Prism 6 software package (GraphPad Software), using two-way ANOVA with Bonferroni’s post-hoc test for multiple comparisons, Student’s t-test or one-way ANOVA followed by Bonferroni’s post-hoc test for multiple comparisons, as indicated. Differences were considered significant at p < 0.05.

### Data availability

Data presented in this study is available from the corresponding author upon request.

### Ethics approval and consent to participate

The WT parent line BIONi010-C was generated by Bioneer from normal adult human skin fibroblasts sourced from Lonza (#CC-2511), who provide the following ethics statement: ‘These cells were isolated from donated human tissue after obtaining permission for their use in research applications by informed consent or legal authorization’.

## Supplementary Information

Supplementary Information.
