# Supplementary material for: PLCγ2 regulates TREM2 signalling and integrin-mediated adhesion and migration of human iPSC-derived macrophages
Source: Sci Rep. 2021 Oct 6;11:19842. doi: 10.1038/s41598-021-96144-7 (PMC8494732; doi:10.1038/s41598-021-96144-7)

SUPPLEMENTARY MATERIAL

PLCγ2 regulates TREM2 signalling and integrin-mediated adhesion and migration of human iPSC-derived macrophages

Juliane Obst[1,*], Hazel L. Hall-Roberts[1,2,+], Thomas B. Smith[1], Mira Kreuzer[3,$], Lorenza Magno[4], Elena Di Daniel[1,#], John B. Davis[1], Emma Mead[1]

[1] Alzheimer's Research UK Oxford Drug Discovery Institute, Centre for Medicines Discovery, University of Oxford, Oxford, UK; [2]James Martin Stem Cell Facility, Sir William Dunn School of Pathology, University of Oxford, Oxford, UK; [3]Department of Oncology, MRC Weatherall Institute of Molecular Medicine, University of Oxford, Oxford, UK; [4]UCL Alzheimer's Research UK, Drug Discovery Institute, London, UK; [+] Present address: UK Dementia Research Institute (UK DRI) at Cardiff University, Cardiff, UK, [$]Present address: Institute of Pathology, University of Munich, Munich, Germany, [#]Present address: Astex Pharmaceuticals, Cambridge, UK

(*) Corresponding author: Juliane Obst. Alzheimer's Research UK Oxford Drug Discovery Institute, Centre for Medicines Discovery, University of Oxford, Oxford, UK. E-mail: juliane.obst@cmd.ox.ac.uk

Supplementary figure 1:

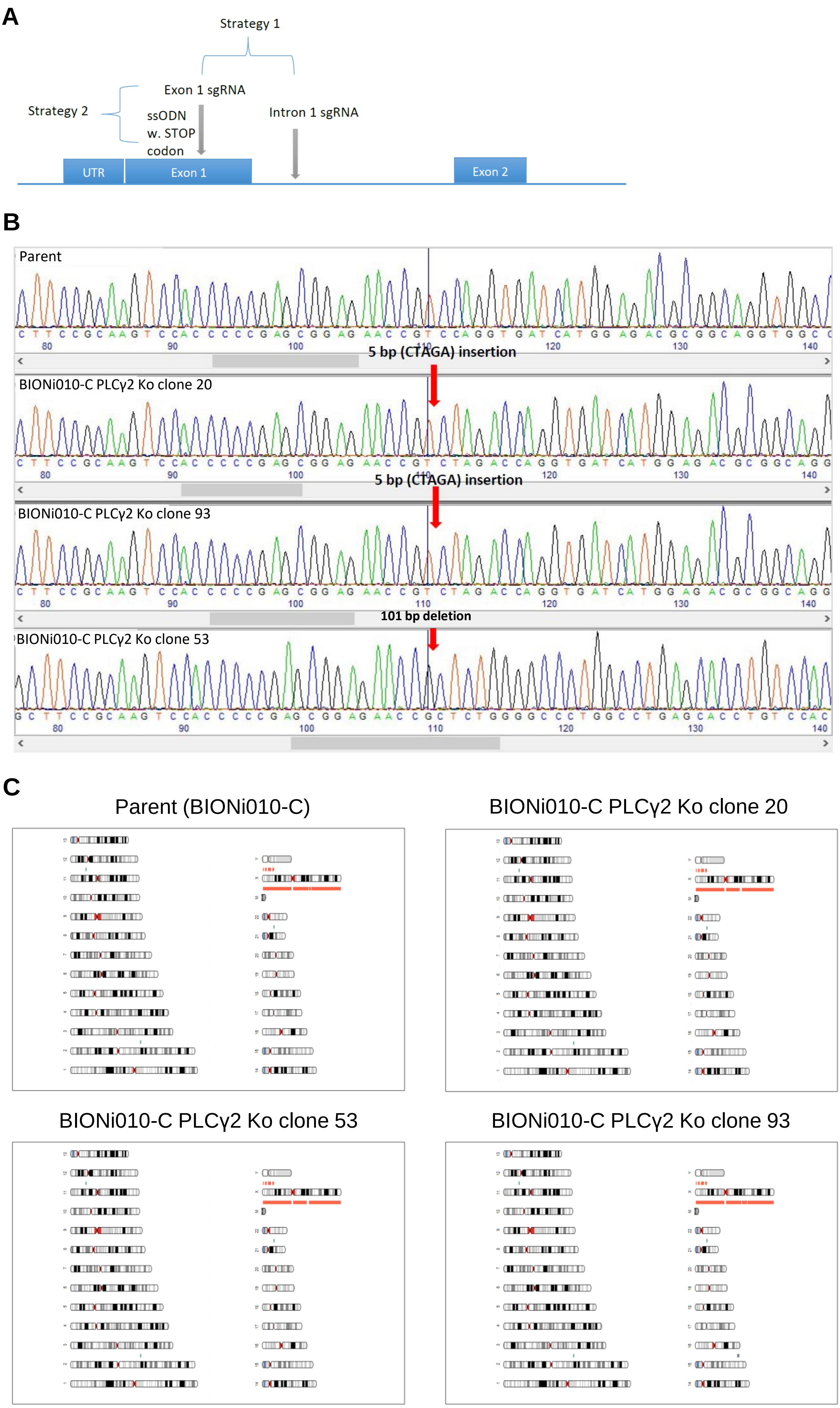

Supplementary figure 1: Generation of genetically modified iPSC lines.
[A] Two strategies were applied that lead to a premature stop of translation: Exon 1 and Intron 1 sgRNA's were used to cut out the last part of Exon 1 in *PLCG2* or Exon 1 sgRNA and a ssODN was used to insert a STOP codon in Exon 1. [B] Sequencing analysis of PLCγ2 Ko clones 20, 53 and 93. The red arrow indicates the site of the CRISPR cutting in the first allele [C] Chromosome karyograms of Parent and PLCγ2 Ko clones 20, 53 and 93 from Illumina microarray SNP analysis.

Supplementary figure 2:

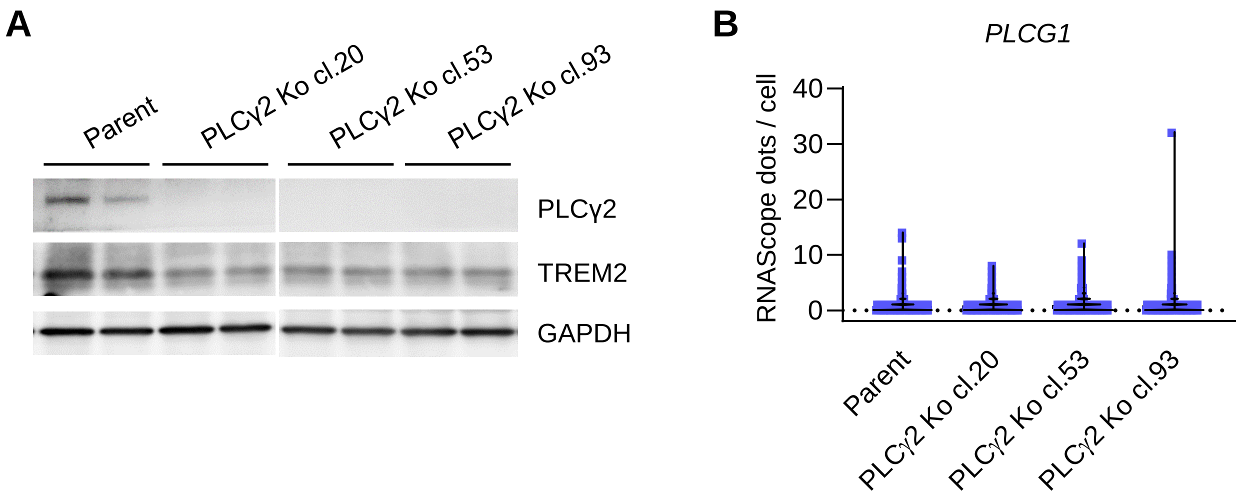

Supplementary figure 2: Validation of PLCγ2 Ko iPSC macrophages clones 20, 53 and 93.
[A] Levels of total PLCγ2 in Parent line and Ko clones. [B] RNAscope analysis of PLCG1 transcripts in Parent vs. Ko lines.

Supplementary figure 3:

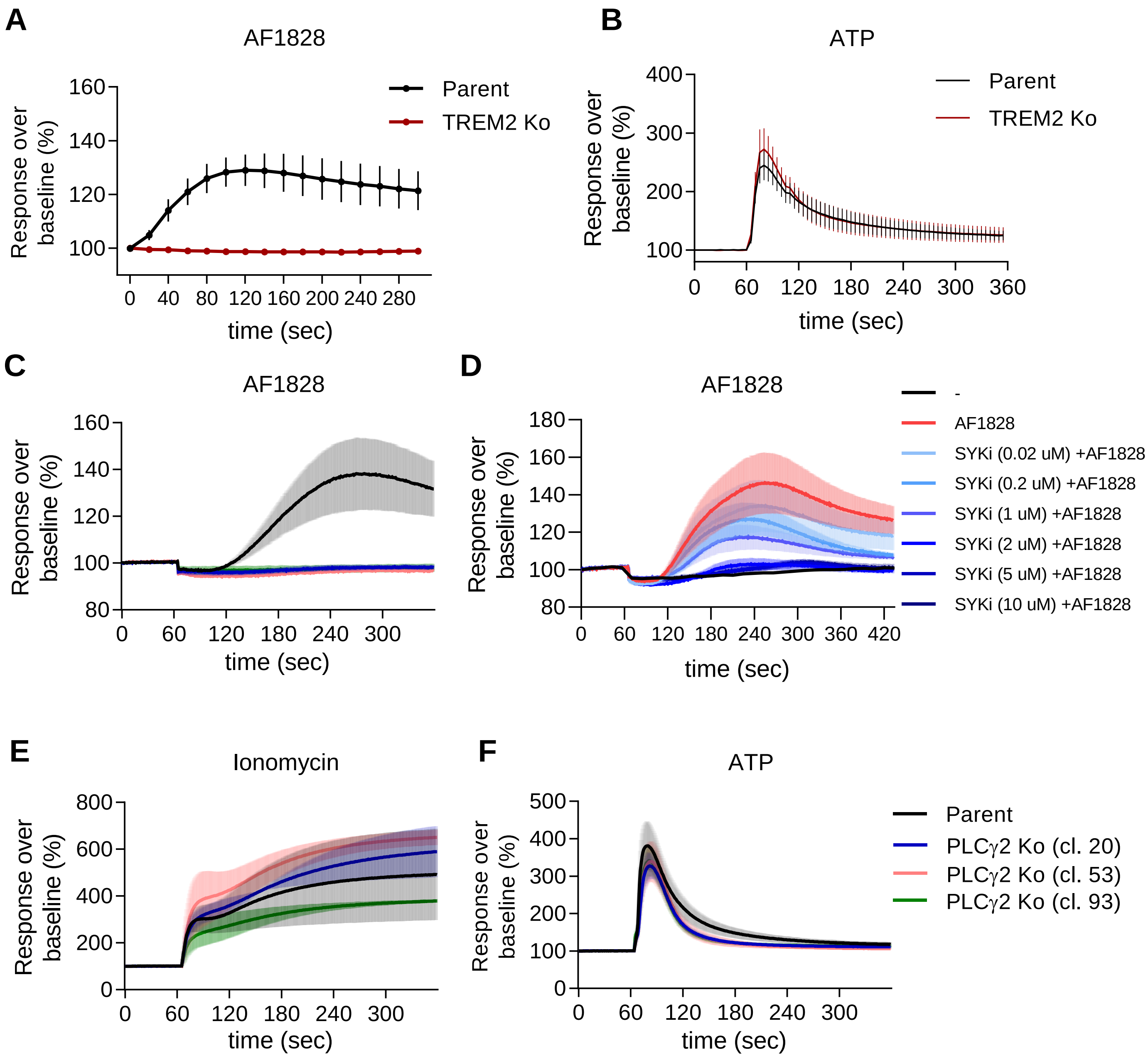

Supplementary figure 3: Characterisation of the Ca2+ assay.
[A] Kinetics of calcium response to TREM2 antibody (AF1828, 5 ug/ml) in Parent and TREM2 Ko line. N=4 [B] Kinetics of calcium response to ATP (0.5 mM) in Parent and TREM2 Ko line. N=4 [C] Calcium kinetics upon stimulation with 5 ug/ml TREM2 antibody in Parent and PLCγ2 Ko (clones 20, 53 and 93). N=3 [D] Concentration dependent inhibition of TREM2- induced calcium response using a SYK inhibitor (BIIB-057). N=3 [E] Kinetics of calcium response to ionomycin (5 uM) in Parent and PLCγ2 Ko clones. N=2 [F] ATP- induced calcium flux in Parent and PLCγ2 Ko clones. n=4, all data shown represent mean ± SEM.

Supplementary figure 4:

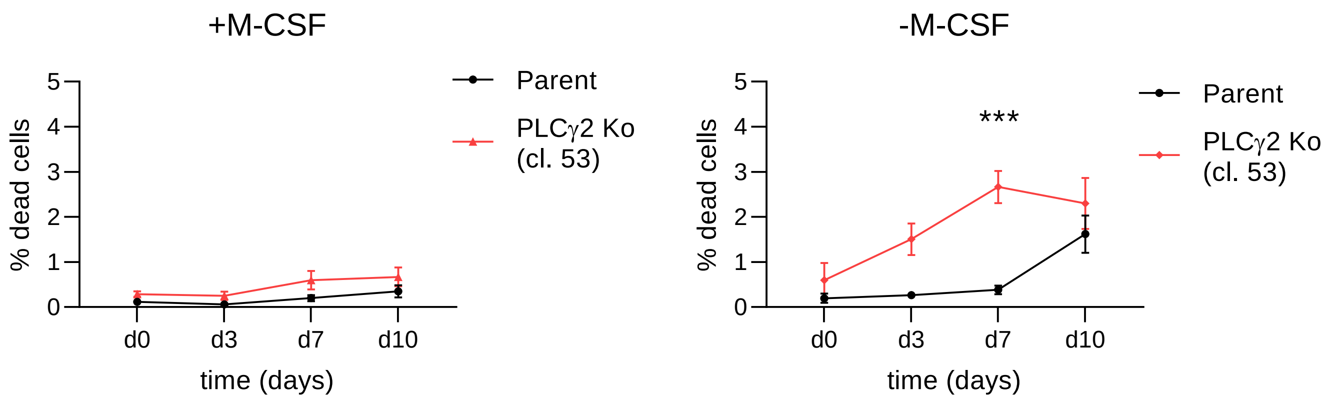

Supplementary figure 4: Survival analysis of PLCγ2 Ko clone 53 in the presence or absence of M-CSF.
Cell death under normal culture conditions (+M-CSF) after ≥ 7 days in the PLCγ2 Ko clone 53 compared to the Parent. In the absence of M-CSF (-M-CSF), PLCγ2 Ko clone 53 showed enhanced sensitivity to cell death than the Parent line. n = 3 , all data shown represent mean ± SEM. two-way ANOVA followed by Bonferroni's multiple comparison test. * $p < 0.05$, ** $p < 0.01$, *** $p < 0.001$

Supplementary figure 5:

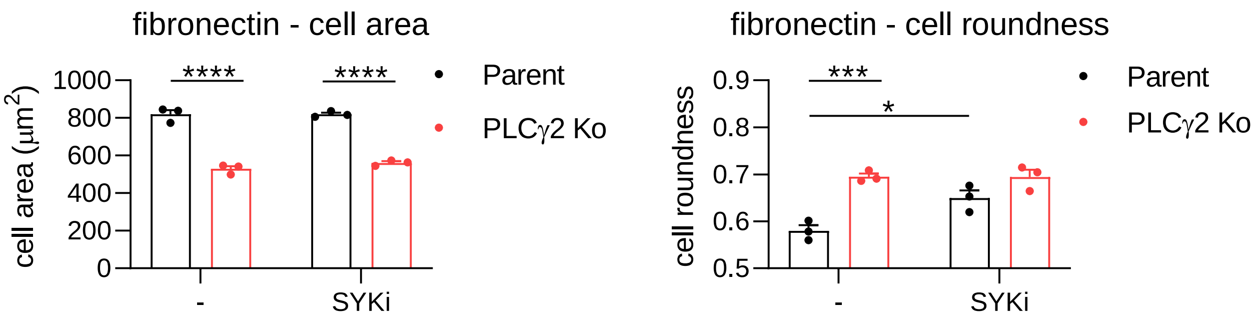

Supplementary figure 5: Effect of PLCγ2 Ko on cell spreading on fibronectin.
Fibronectin coating does not rescue cell area or cell roundness in PLCγ2 Ko cells. N=3, all data shown represent mean ± SEM. two-way ANOVA followed by Bonferroni's multiple comparison test. * $p < 0.05$, ** $p < 0.01$, *** $p < 0.001$

Supplementary figure 6:

This figure shows the full original uncropped Western blot images of Figure 1A (results)

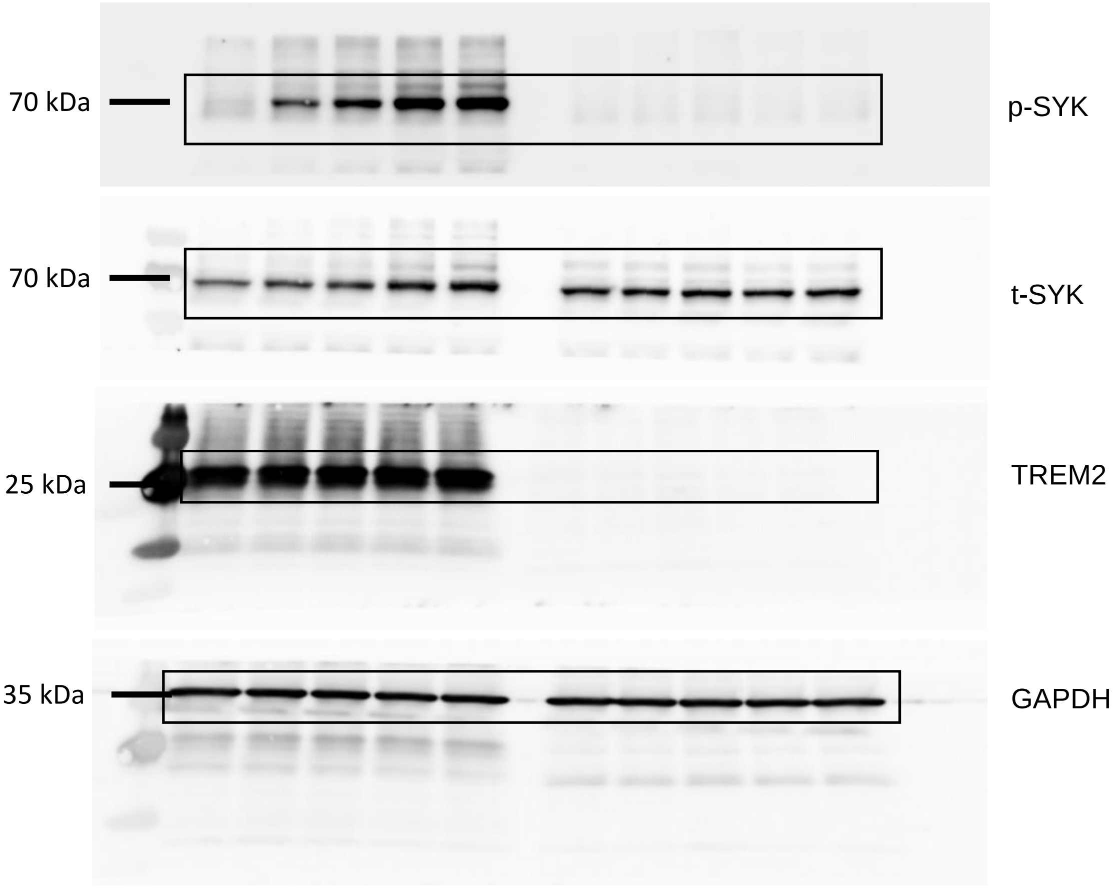

Supplementary figure 7:

This figure shows the full original uncropped Western blot images of Figure 2A (results)

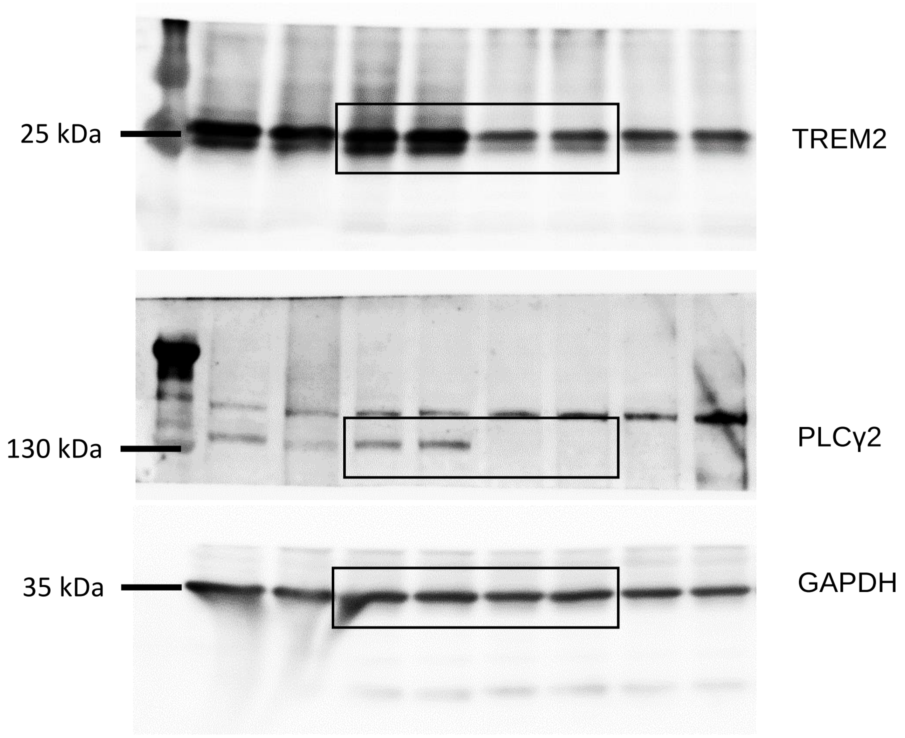

Supplementary figure 8:

This figure shows the full original uncropped Western blot images of Suppl. Figure 2

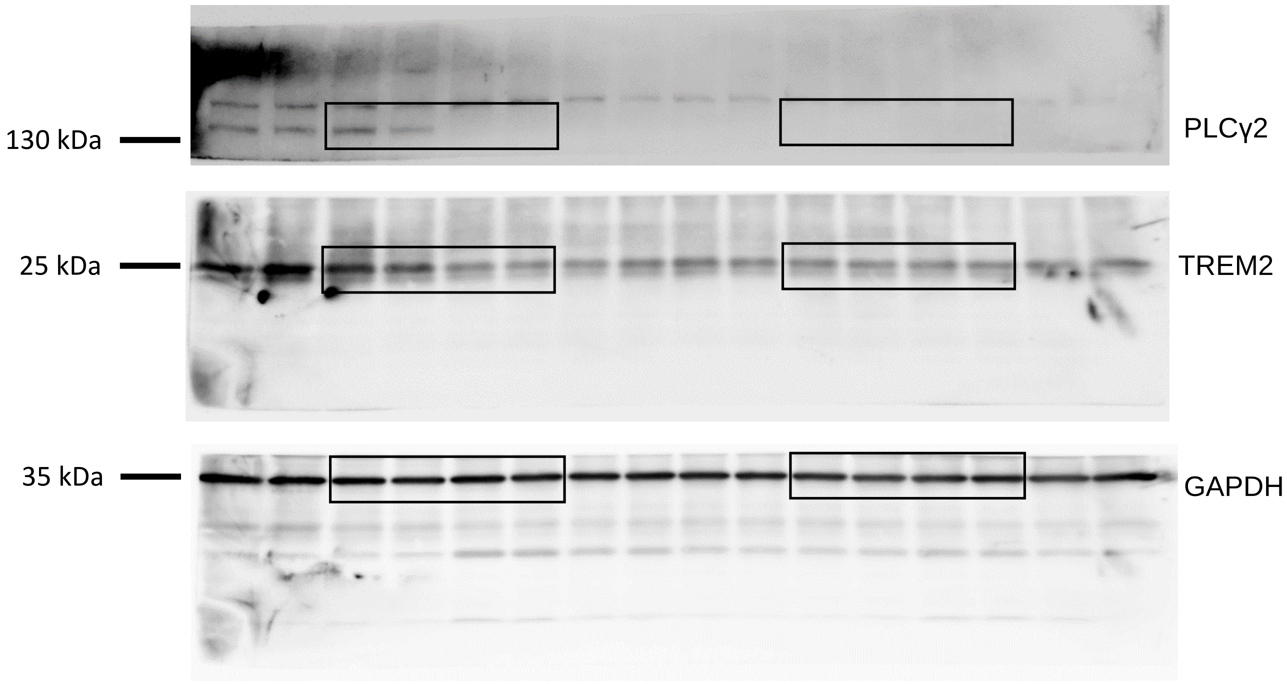

Supplementary figure 9:

This figure shows the full original uncropped Western blot images of Figure 3A (results)

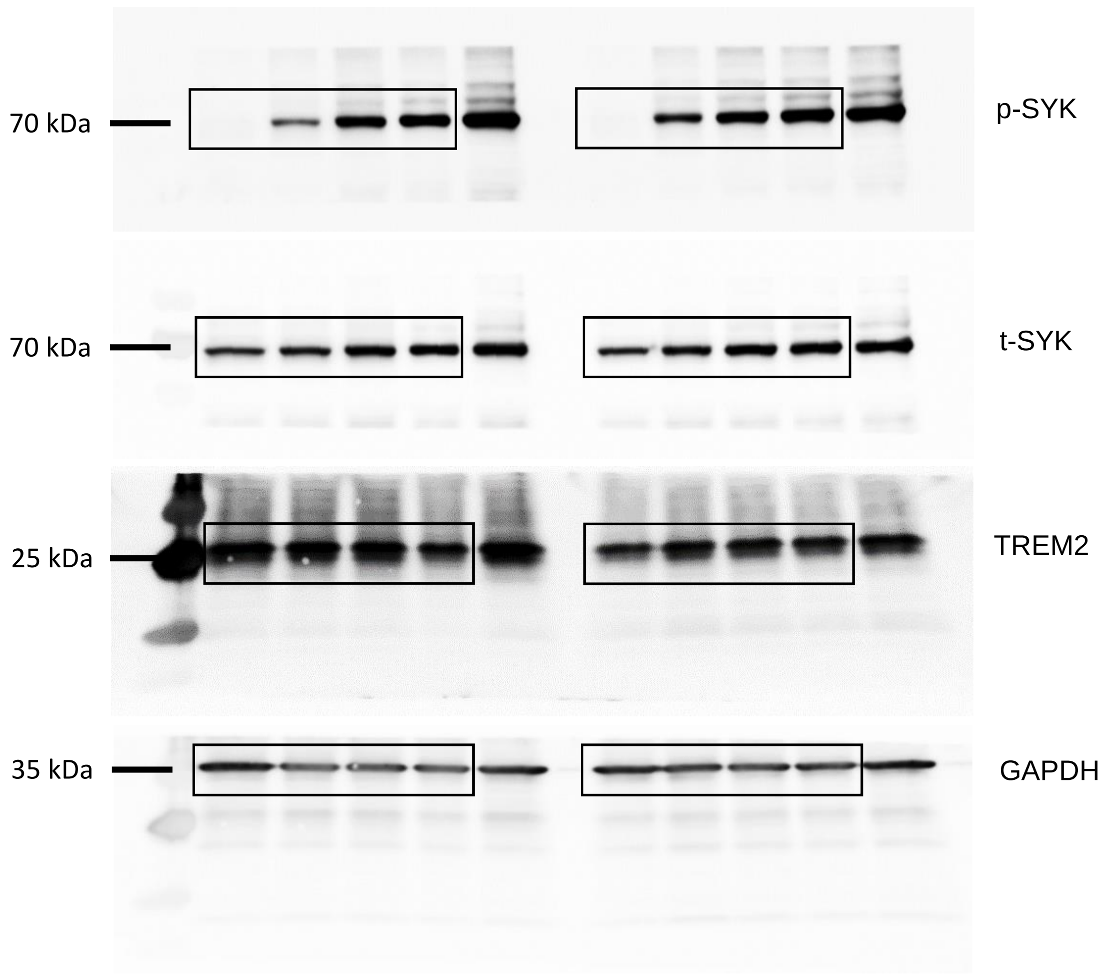

Supplementary figure 10:

This figure shows the full original uncropped Western blot images of Figure 3F (results)

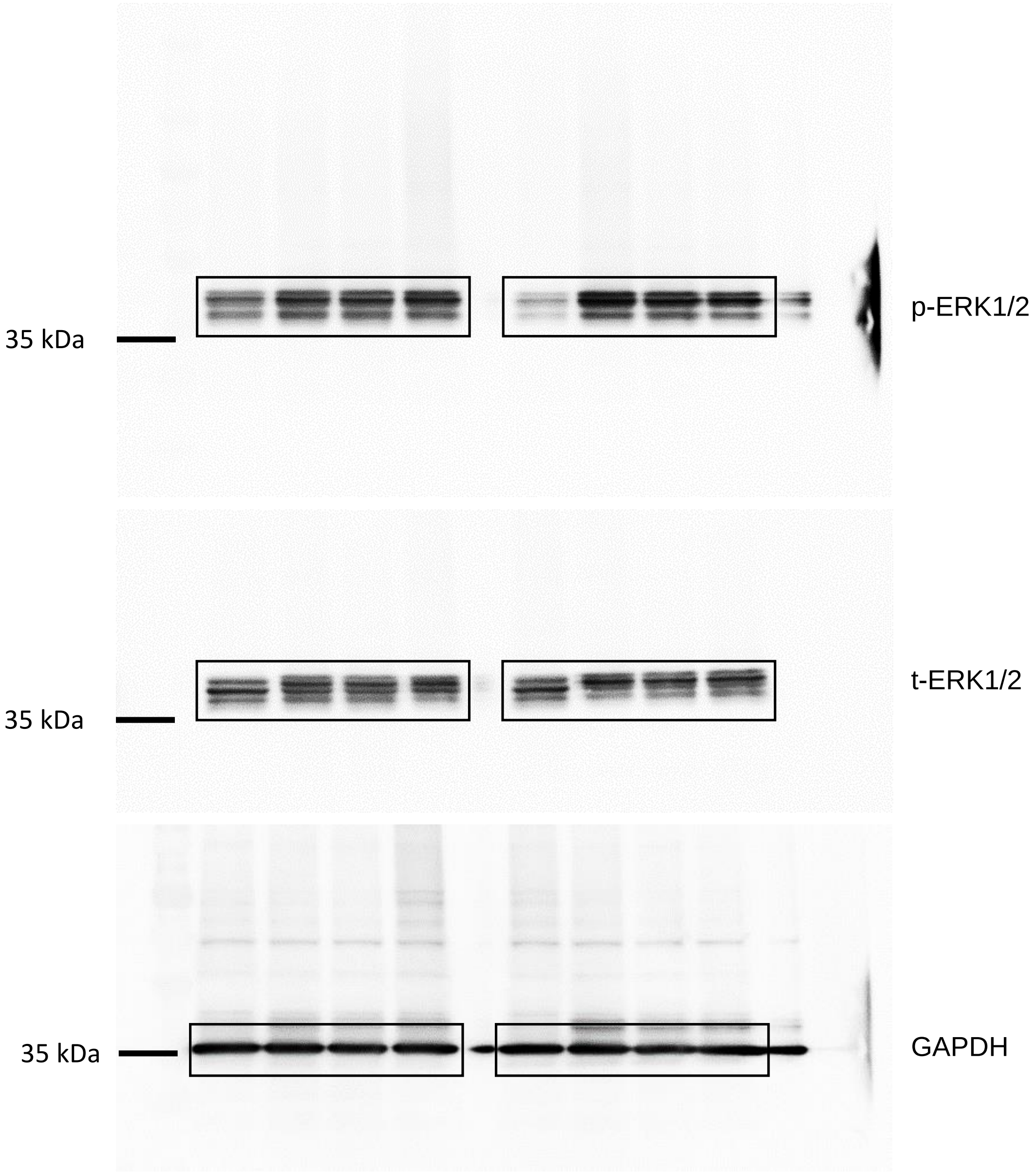

Supplementary figure 11:

This figure shows the full original uncropped Western blot images of Figure 5E (results)

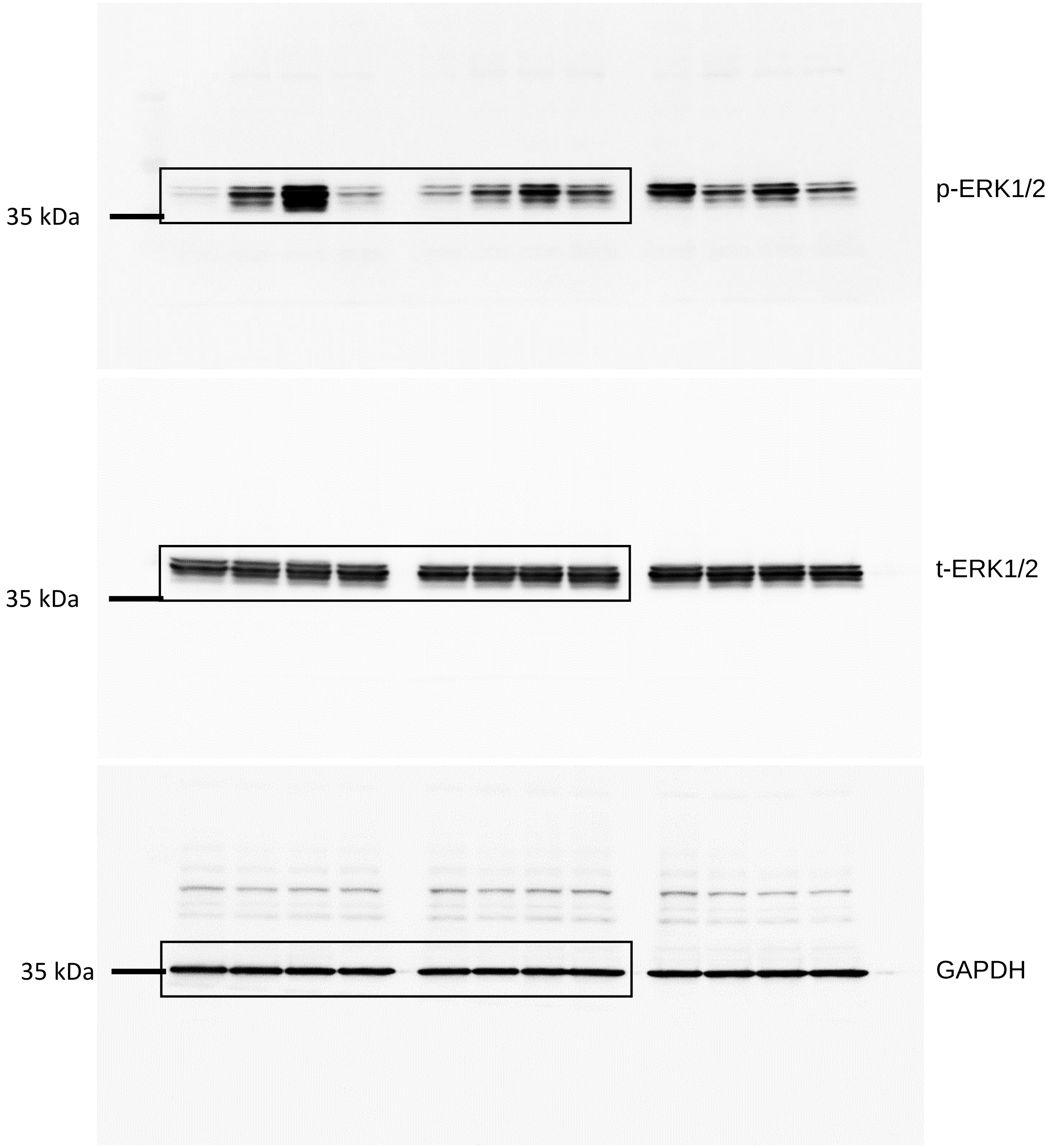

Supplement: Supplementary file 1 — Supplementary Information. [file 41598_2021_96144_MOESM1_ESM.pdf]
